# The identity and role of the radiologist in 2020: a survey among ESR full radiologist members

**DOI:** 10.1186/s13244-020-00945-9

**Published:** 2020-12-03

**Authors:** European Society of Radiology (ESR), Andrea Rockall, Adrian P. Brady, Lorenzo E. Derchi

**Affiliations:** European Society of Radiology (ESR), Am Gestade 1, 1010 Vienna, Austria

**Keywords:** Education and training, Professional issues, Patient communication, Job satisfaction

## Abstract

**Background:**

Radiologists undertake a wide variety of functions which have altered as technologies have evolved. The aim of this survey was to explore radiologists’ opinions concerning their role and identity in 2020.

**Methods:**

The survey included 124 questions on training, daily work, interaction with colleagues and patients, involvement in teaching, research and management and task delegation. An initial draft was amended following responses from the Presidents of the 48 ESR’s national institutional member societies. The final on-line survey was available to individual ESR full members between January and March 2020. 1344 responses from radiologists in 49 European countries were obtained.

**Findings:**

80% (1049/1317) of radiologists considered a period of non-radiology clinical training mandatory and 92% (1192/1291) felt that sub-specialty expertise was important and improved the visibility of radiologists. 76% (961/1262) of radiologists regularly communicate directly with patients. Only 25% (314/1238) had undergone formal communications training although 82% (1020/1238) felt that this would be beneficial. Radiologists highly value their positive interaction with colleagues, including within multi-disciplinary team meetings, despite limited resources. Difficulties identified included high workload, especially the need to cover general work in parallel with the need to offer subspecialty expertise. 66% (837/1262) felt that lack of visibility to patients is a risk to radiology and professional visibility could be improved by radiology-led research and teaching.

**Conclusions:**

ESR activities should aim to (1) support radiologists with sub-specialty training and maintenance of competencies; (2) develop recommendations for patient communications training and multi-disciplinary working with strong clinical integration; (3) enhance radiologists’ visibility by harnessing opportunities for radiology-led research and education.

## Main messages

- Society activities should continue to support clinical and sub-specialty training and accreditation.
- A communications strategy should be developed that includes a range of resources for patient information and radiology-specific communications training.
- Guidance may be helpful for integration of multi-disciplinary team working, especially in the context of limited resources.
- The visibility of the specialty should be enhanced by highlighting radiology-led research and teaching.

## Patient summary

*Training and Practice* Radiologists see their role as highly clinical. Most radiologists consider it important to have experience in non-radiological clinical care and to have a sub-specialty expertise within radiology, despite recognising the importance of the general workload.

*Patient and professional relationships* 75% of respondents regularly interact with patients face-to-face. Among those who do not, 43% would prefer having some opportunity to do so. Around half of respondents have some opportunity to go through radiology findings with patients but only 25% feel that there is sufficient time to discuss. Only 25% of respondents underwent formal patient communication training, although 83% felt that radiologists should undertake such training to communicate bad news and imaging results.

Communicating errors to referring clinicians and to patients was considered important or very important (79%). This is a difficult but essential task as reviewing errors is considered crucial learning and shaping better specialists and humans.

Radiologists’ input to Multi Disciplinary Team work is valued, and 56% of respondents already have the key role of integrating their information about the patient (clinical, imaging, pathology, outcomes, follow-up).

Overall, 66% of respondents felt that the lack of visibility to patients was a risk to the radiology profession and improvements needed to be considered.

*Teaching, research and management* Most respondents (69%) have never received any formal “training to teach”, although 73% think this would be important or very important. A role in teaching was largely considered to improve the visibility of radiologists to both the institution and to clinical colleagues.

Respondents have a high (32%) or very high (25%) level of interest in taking part in research. However, opportunities are not always available. Most respondents feel that research led by radiologists is important (42%) or very important (41%) to their identity as a profession and improves visibility to clinical colleagues (91.5% agree or totally agree), to patients and to the media.

Opportunities for management and service development are relatively limited. An optional survey section which looked at areas of delegation to radiographers was completed by most respondents with a wide range of opinions.

The paper highlights several recommendations for consideration by the ESR and its members in response to the findings of the survey.

## Background and objectives

Although the core work of a radiologist as a diagnostician and/or interventionalist is widely recognised in the medical community, radiologists are often regarded by the lay public as “invisible readers of images” and the Radiology Department is often considered, especially by healthcare administrators, as an “examination factory”, and not as a clinical service.

Our work entails much more than simply “reading” and has many other strands and human factors. An important and often underestimated aspect of our daily work is our relationships with patients, among ourselves, and with other colleagues. Face-to-face patient encounters (e.g. in interventional radiology and ultrasound) facilitate a direct relationship with patients. In addition, there is increasing interest from many radiologists and patient groups in direct delivery of information by radiologists to patients before studies, and provision of reports directly to patients, with explanation of the imaging findings; these represent opportunities for increased radiologist-patient interaction. Relationships with non-radiology colleagues are clear in our routine work, when we provide answers through imaging to the clinical questions given by them when requesting a study. These relationships are fully exploited during Multidisciplinary Team (MDT) Meetings, with discussion of patients’ care involving many different specialists, leading to collective, fully-informed decision-making about therapeutic management. This survey attempts to explore radiologists’ opinions in relation to their own role and their interactions with professional colleagues and patients.

Furthermore, since radiologists undertake many varied tasks outside the core work of diagnostic and interventional radiology, we hoped to explore the range and extent of these additional roles, as well as changes to the roles of allied professionals in our departments.

The final goal was to try to understand our sense of professional identity as radiologists: who do we think we are and where do we fit in current day clinical practice? How do we ensure visibility of radiologists, in order to attract the brightest and the best doctors and ensure the future of our specialty?

## Methods

A draft survey was created by the Chair of the ESR National Societies Committee and circulated to the ESR Executive Council in September 2019. Following revision, the survey was created on “Survey Monkey” and circulated to the Presidents of the European National Societies of Radiology in October 2019. The survey comprised 105 questions in total, and incorporated two optional sections, one regarding interventional radiology (6 questions) and one regarding delegation of tasks in the department (13 questions).

The survey had four main themes:

1. About you, your training and your clinical role.
2. About your patient and professional relationships.
3. About your role in education, research, management and service development.
4. Areas of delegation to allied professionals: what happens in your department and what is your opinion?

The questions included in the full survey are available in supplementary data (Additional file 1: Appendix 1).

The results of the initial survey were presented and discussed at the ESR Annual Leadership Meeting in Genoa in November 2019. Following discussion and written feedback, the survey was amended, all ESR full members were invited by email to participate, and the survey was open online from January 13, 2020 to March 2, 2020.

The key survey results were prepared for the June 2020 ESR National Societies meeting held on-line due to the COVID-19 pandemic. The survey findings were discussed and the conclusions and recommendations were proposed.

## Results

Responses were available from 1344 radiologists, from 49 countries (Table 1). Just over half (54%) worked in academic institutions, 23% in non-university hospitals with a teaching component, 11% in district general or community hospitals without a teaching component and 10% in private practice.

**Table 1:** In which country do you practice? (responses *n* = 1344)

Answer choices	Responses	
Albania	0.74%	10
Andorra	0.07%	1
Armenia	0.60%	8
Austria	1.79%	24
Azerbaijan	0.30%	4
Belarus	0.37%	5
Belgium	2.53%	34
Bosnia and Herzegovina	1.04%	14
Bulgaria	0.67%	9
Croatia	0.97%	13
Cyprus	0.45%	6
Czech Republic	0.74%	10
Denmark	0.89%	12
Estonia	0.45%	6
Finland	1.56%	21
France	2.75%	37
Georgia	0.52%	7
Germany	4.32%	58
Greece	5.43%	73
Hungary	1.41%	19
Iceland	0.52%	7
Ireland	0.37%	5
Israel	0.45%	6
Italy	11.24%	151
Kazakhstan	0.37%	5
Kosovo	0.22%	3
Kyrgyzstan	0.00%	0
Latvia	0.30%	4
Lithuania	0.52%	7
Luxembourg	0.07%	1
Malta	0.15%	2
Moldova	0.07%	1
Monaco	0.00%	0
Montenegro	0.15%	2
The Netherlands	3.35%	45
North Macedonia	0.22%	3
Norway	2.08%	28
Poland	1.71%	23
Portugal	1.56%	21
Romania	2.98%	40
Russian Federation	2.16%	29
Serbia	1.34%	18
Slovakia	0.60%	8
Slovenia	0.67%	9
Spain	10.27%	138
Sweden	2.98%	40
Switzerland	2.16%	29
Turkey	14.73%	198
Ukraine	1.71%	23
United Kingdom	9.23%	124
Uzbekistan	0.22%	3

### Theme 1: About you, your training and your clinical role.

**Fig. 1:**
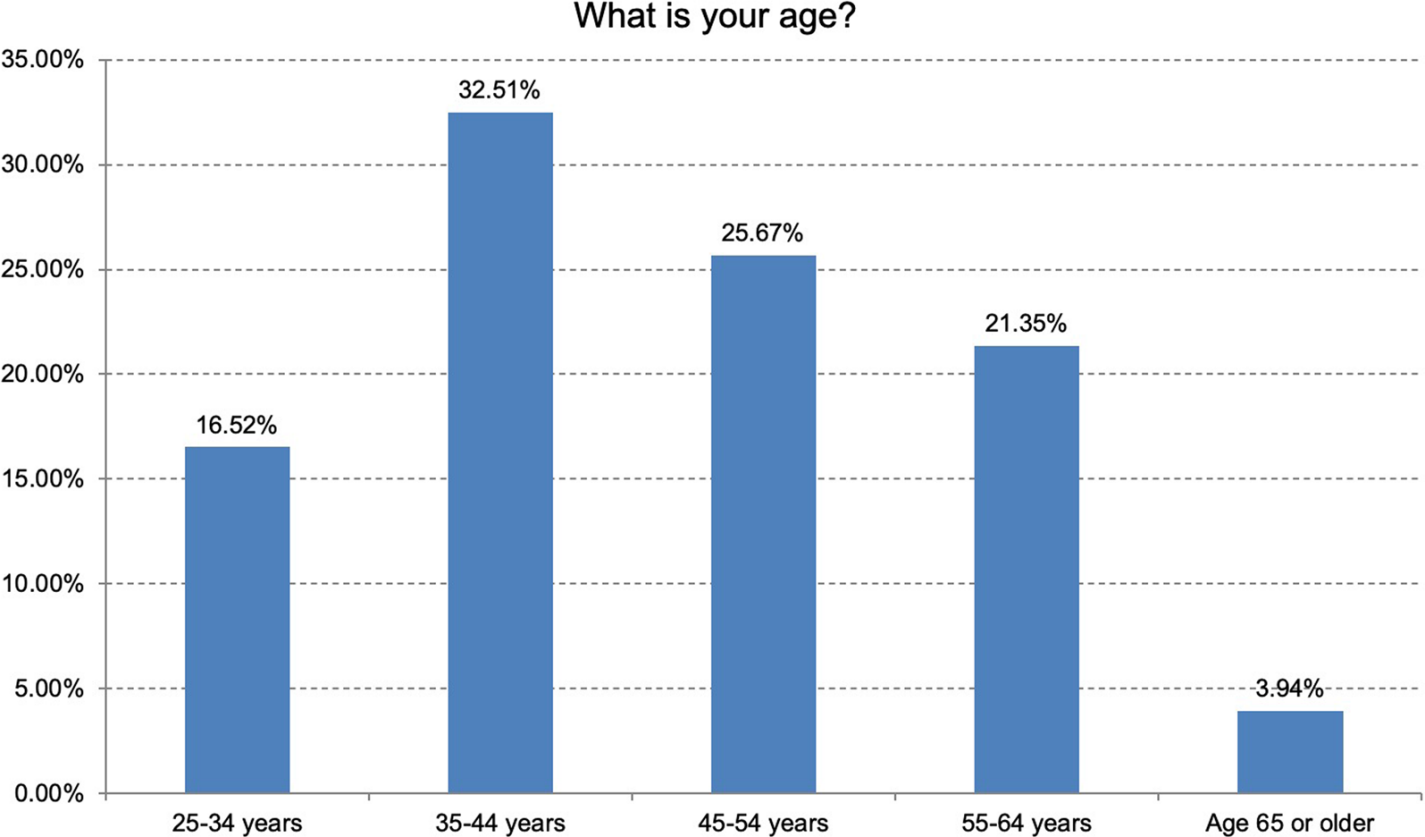
The age range of respondents (responses *n* = 1344)

**Fig. 2:**
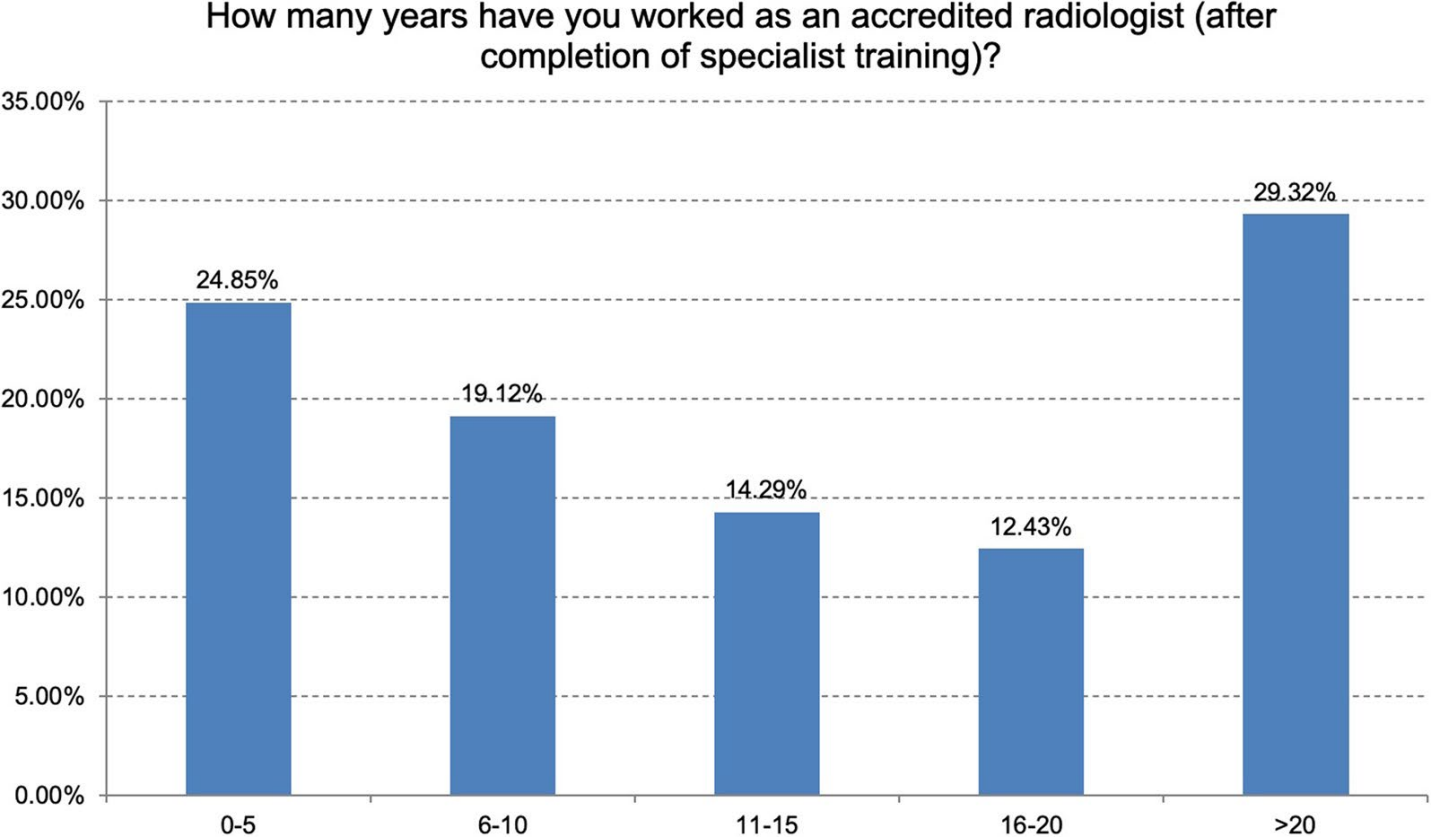
The time working as a fully accredited radiologist (responses *n* = 1344)

33% of respondents were aged between 35 and 44 years, 26% between 45 and 54 years; 57% were male. 44% had been in practice for up to 10 years, 27% for 11–20 years and 29% for over 20 years (Figs. 1, 2). Part-time working was available for women in 60% and for men in 56% of departments. Approximately half of departments offered part-time or flexible working arrangements, slightly more commonly available to women than to men.

#### Training

Non-radiological clinical training had been undertaken by 70%, most commonly for over 24 months (41%) or between 12 and 24 months (20%). Clinical training was undertaken as a radiology resident in approximately 55%, as a clinical resident in 40% and as a medical student in 28%. 80% of respondents said that a period of non-radiology clinical experience should be mandatory, either before or during radiology training; the most-selected duration was 9–12 months.

Research during training had been undertaken by obtaining a research degree (MSc or PhD) in 33%. Research was undertaken informally as part of training by 27%, and no research was undertaken during radiology training by 40%. Undertaking research as part of radiology training was a requirement in 30% of training programmes.

Subspecialty training had been undertaken by 47% of respondents, with a wide range of time in sub-speciality training, ranging from 3 to 6 months (28%) to over 24 months (18%), 12–24 months being most typical (32%). Two thirds had undertaken sub-speciality training in a different department to their base training department, suggesting that this experience also provided a wider exposure to different ways of working.

**Fig. 3:**
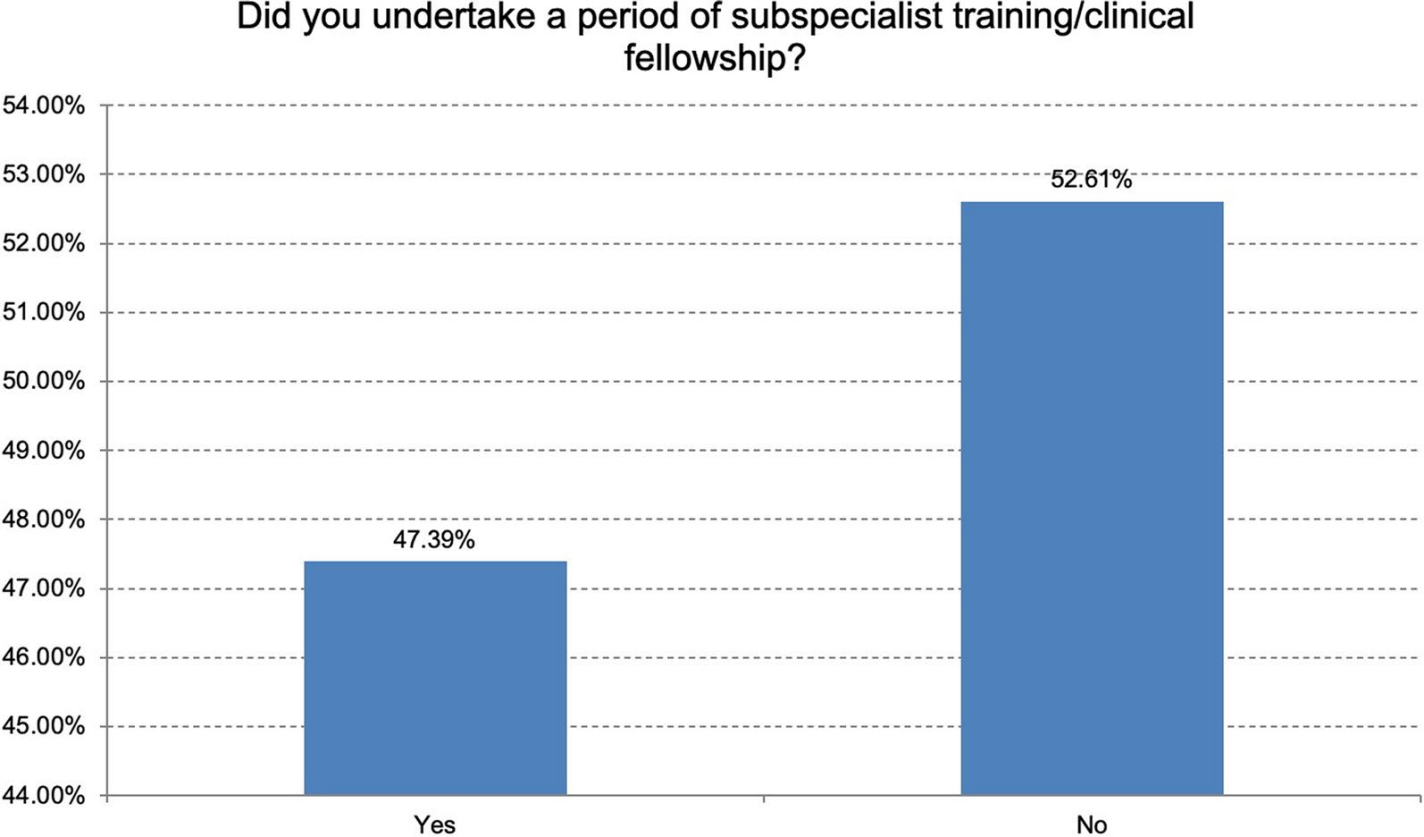
The percentage of respondents that had undertaken sub-specialty training (responses *n* = 1302)

**Fig. 4:**
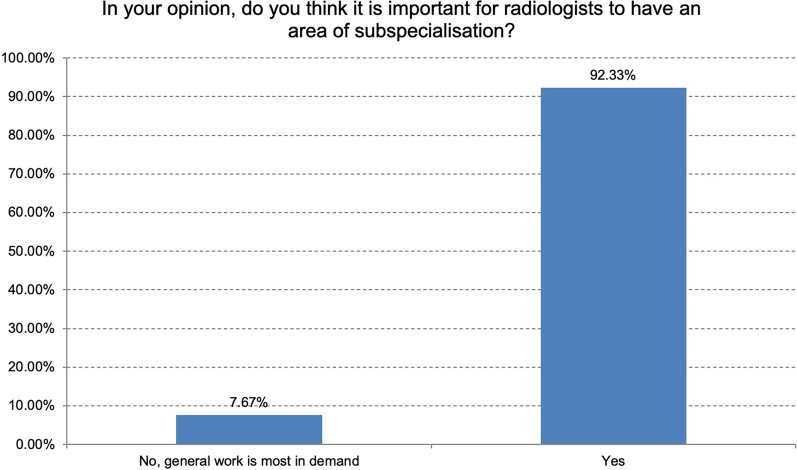
The percentage of respondents that think sub-specialty training is important (responses *n* = 1291)

When asked whether having an area of sub-specialisation was important, 92% of respondents said yes, with less than 10% selecting the response that general work is most in demand (Figs. 3, 4). There were 306 individual written comments concerning this topic, with many reflecting the tension between the need to cover the general clinical service against the need to offer a sub-specialist expert opinion, to contribute to excellence in patient care in highly complex imaging scenarios.

**Fig. 5:**
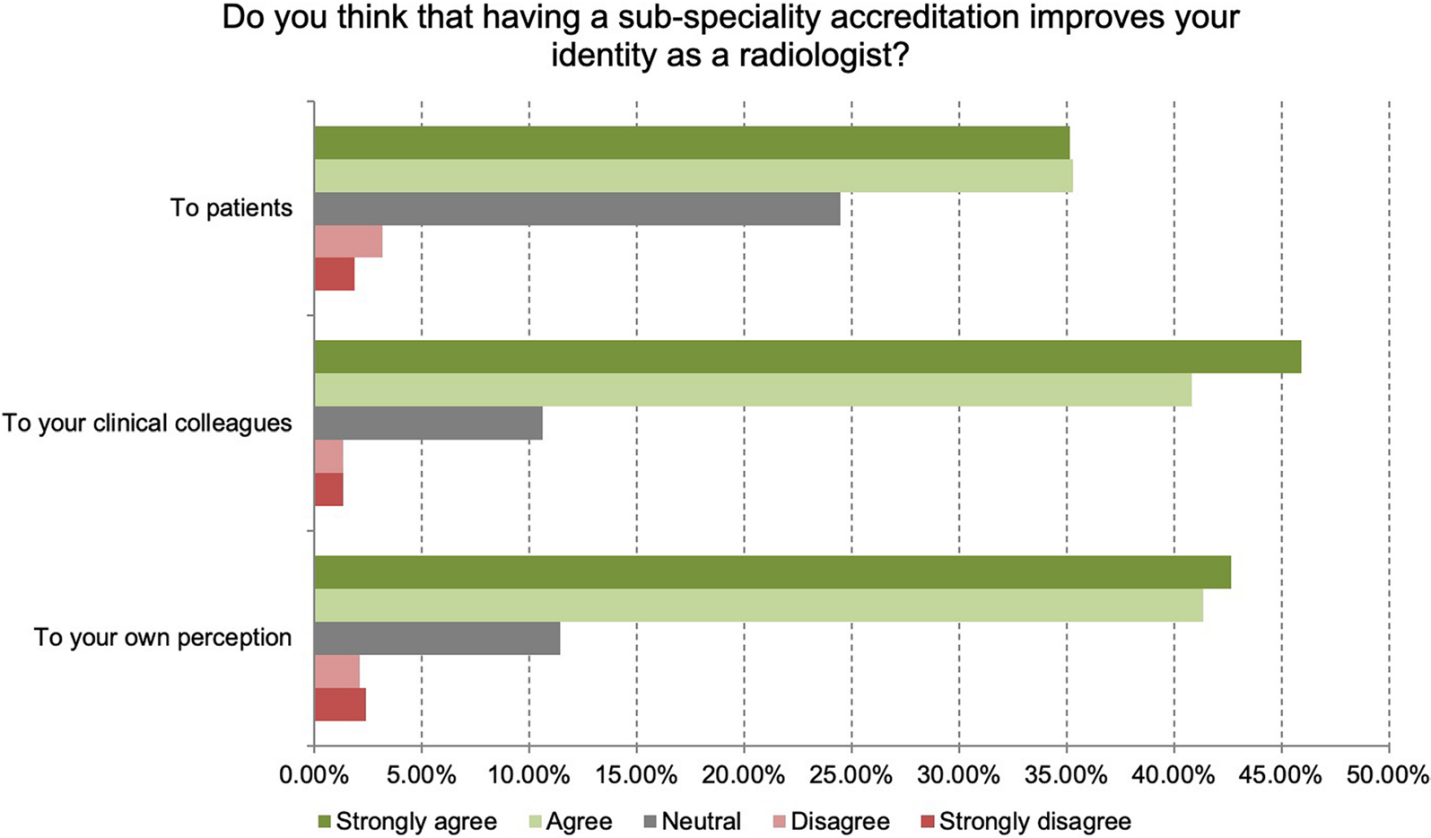
Do you think that having a sub-speciality accreditation improves your identity as a radiologist? (responses *n* = 1291)

Sub-specialty accreditation was thought to improve the respondent’s identity as a radiologist, mainly in relation to other clinical colleagues (87% agreed or strongly agreed) but also to themselves as a radiologist (84% agreed or strongly agreed). (Fig. 5).

**Fig. 6:**
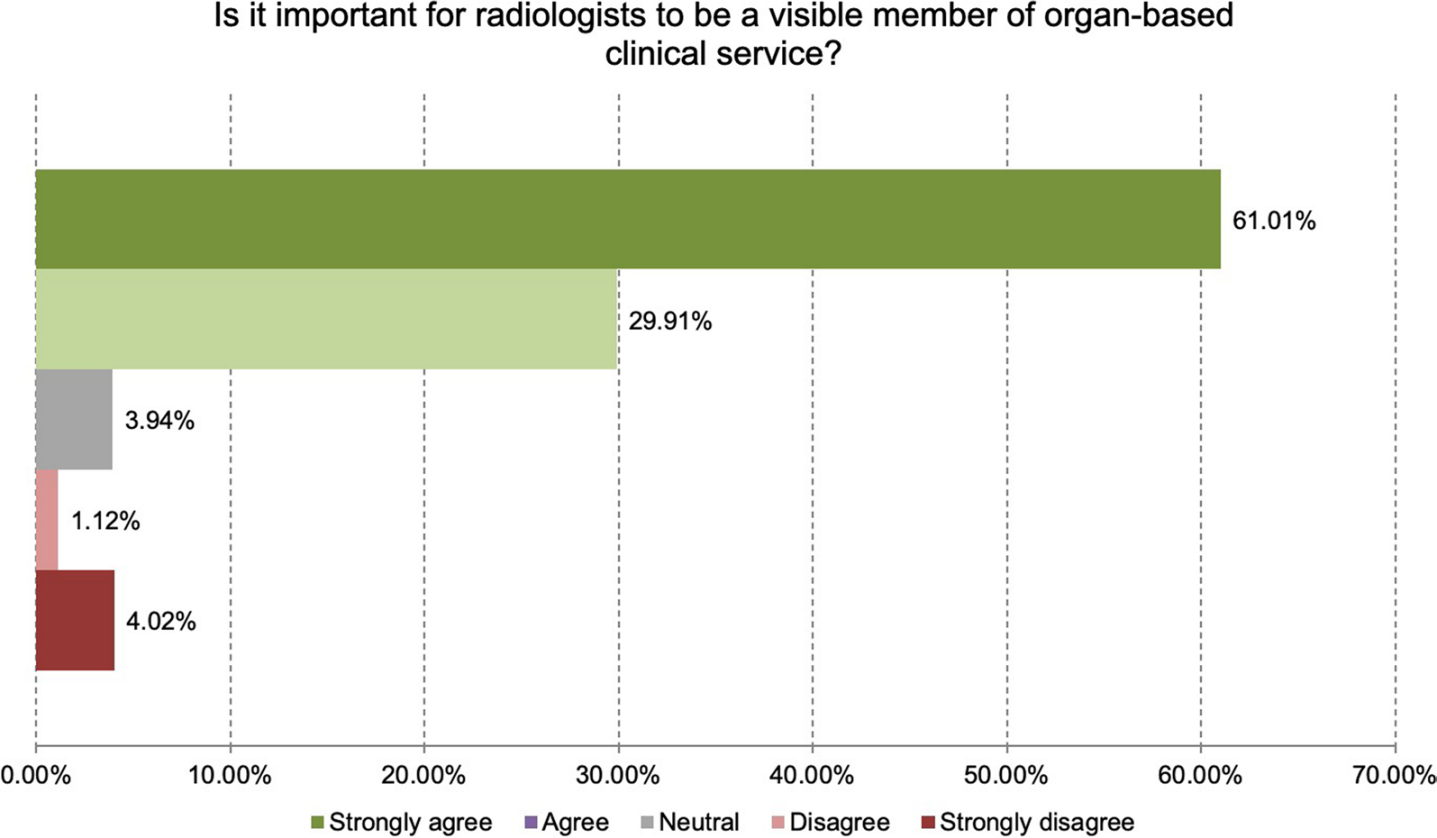
In your opinion, is visibility important for our identity in the clinical service e.g. is it important for radiologists to be a visible member of organ-based clinical service? (responses *n* = 1344)

It was felt that being a visible member of an organ-based clinical service was important to our identity as a radiologist (61% strongly agree, 30% agree, Fig. 6).

Informatics and AI training, including computing technology and/or software engineering skills, should be provided at a basic level in the core radiology curriculum at a basic level, according to 60% of respondents. However, 37% suggested that these skills should be provided at an advanced level during core training.

#### Role in clinical practice

**Fig. 7:**
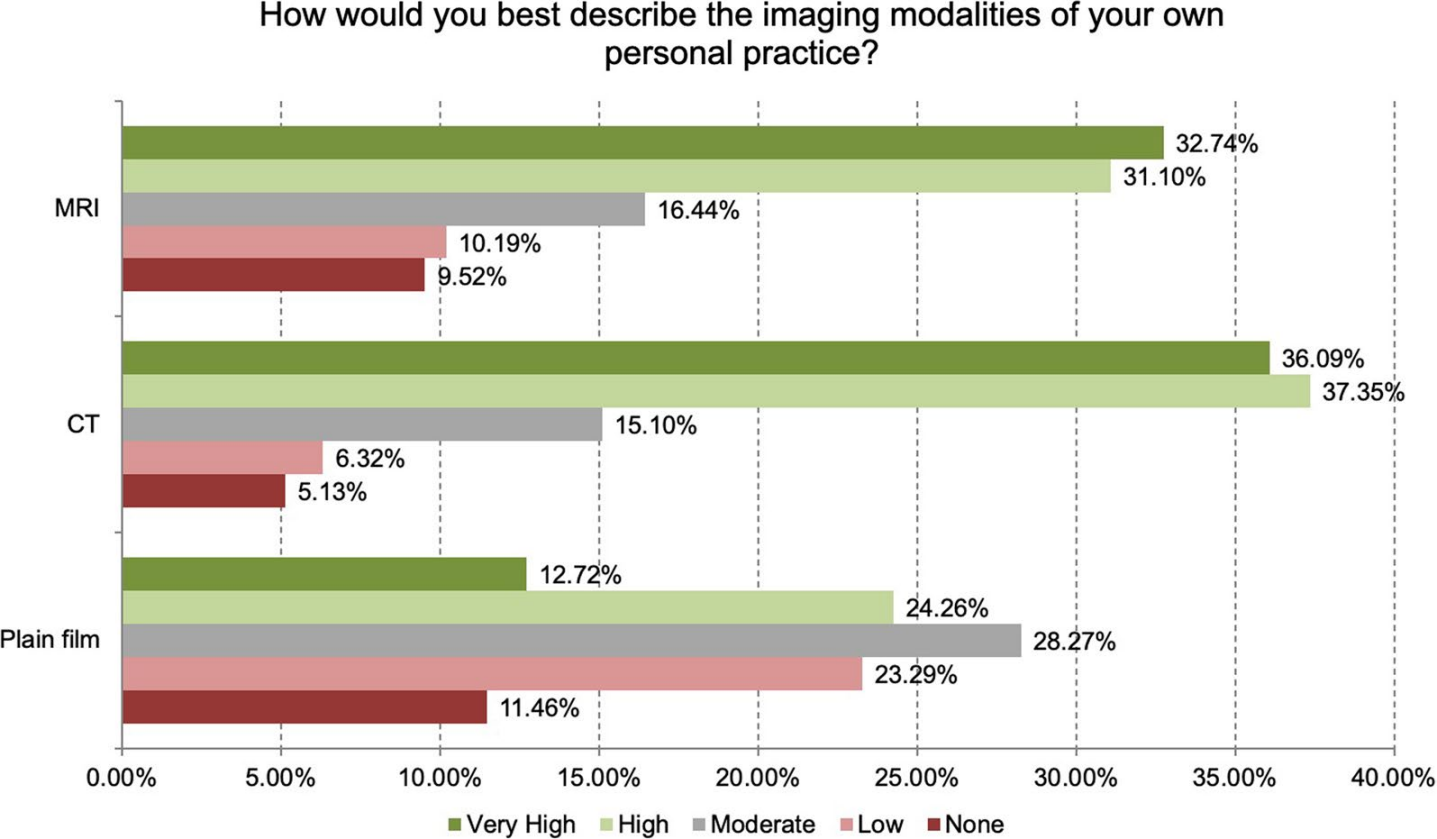
Range of modalities used in the respondents’ personal practice (responses *n* = 1344)

Respondents were asked to describe the distribution of modalities that they use in their own clinical practice (Fig. 7).

The top three modalities practiced by respondents were CT (high or very high use in 73%), MRI (high or very high use in 64%) and ultrasound (high or very high use in 55%) (Table 2). Only 5% of respondents do not use CT in their practice.

**Table 2:** How would you best describe your own "organ-based or clinical" areas of practice? (responses *n* = 1344)

	None		Low		Moderate		High		Very high	
General	6.99%	94	10.94%	147	30.65%	412	36.68%	493	14.73%	198
Neuro	16.74%	225	20.76%	279	29.84%	401	19.20%	258	13.47%	181
Head and Neck	13.17%	177	27.83%	374	34.45%	463	16.00%	215	8.56%	115
Pulmonary	9.90%	133	18.82%	253	31.10%	418	29.39%	395	10.79%	145
Cardiac	44.05%	592	26.93%	362	14.73%	198	8.11%	109	6.18%	83
Breast	43.38%	583	15.10%	203	15.18%	204	13.32%	179	13.02%	175
Gastrointestinal	8.63%	116	11.76%	158	27.98%	376	33.04%	444	18.60%	250
Genitourinary	9.97%	134	12.87%	173	28.50%	383	31.77%	427	16.89%	227
Musculoskeletal	11.61%	156	27.90%	375	24.55%	330	21.73%	292	14.21%	191
Paediatric Imaging	22.17%	298	34.23%	460	25.22%	339	11.61%	156	6.77%	91
Oncology imaging	5.06%	68	8.18%	110	23.51%	316	34.23%	460	29.02%	390
Vascular intervention	67.19%	903	13.54%	182	7.22%	97	4.91%	66	7.14%	96
Non-vascular intervention	37.05%	498	20.68%	278	20.46%	275	12.28%	165	9.52%	128

Plain film work was most commonly moderate (28% moderate, 24% high, 13% very high). Some radiologists do not report plain films (11.5%).

Respondents were asked to describe their organ-based or clinical areas of practice. The highest selected response was oncology followed by both gastro-intestinal and general radiology (with the same score), then genito-urinary and pulmonary imaging (Table 2).

When asked what aspects of working as a radiologist they like most, the highest-ranking response was being a specialist radiologist, closely followed by interaction with radiology and clinical colleagues and MDT meetings. The lowest ranked aspects were being a generalist radiologist, audit and service evaluation, with the lowest being interaction with hospital management (Table 3).

**Table 3:** How do you rate your enjoyment of different aspects of your job? (responses *n* = 1344)

	Strongly disagree		Disagree		Neutral		Agree		Strongly agree		Not applicable	
Being a general radiologist	4.02%	54	8.26%	111	21.13%	284	36.24%	487	25.30%	340	5.06%	68
Being a specialised radiologist	0.67%	9	2.01%	27	7.59%	102	23.29%	313	64.06%	861	2.38%	32
Interaction with colleagues in the radiology department	0.74%	10	1.56%	21	8.41%	113	29.46%	396	58.48%	786	1.34%	18
Interaction with clinical colleagues	0.37%	5	2.08%	28	9.45%	127	31.92%	429	54.91%	738	1.26%	17
Involvement in multidisciplinary meetings/tumour board	1.49%	20	3.27%	44	11.24%	151	28.57%	384	50.45%	678	4.99%	67
Interaction with patients	1.19%	16	4.46%	60	18.60%	250	39.96%	537	34.23%	460	1.56%	21
Teaching	1.49%	20	2.75%	37	15.33%	206	34.52%	464	40.40%	543	5.51%	74
Service evaluation and audit	4.17%	56	11.68%	157	32.66%	439	30.65%	412	13.76%	185	7.07%	95
Performing research	3.79%	51	9.30%	125	22.17%	298	29.91%	402	26.64%	358	8.18%	110
Involvement with hospital management	7.66%	103	17.04%	229	29.84%	401	23.88%	321	14.06%	189	7.51%	101

**Fig. 8:**
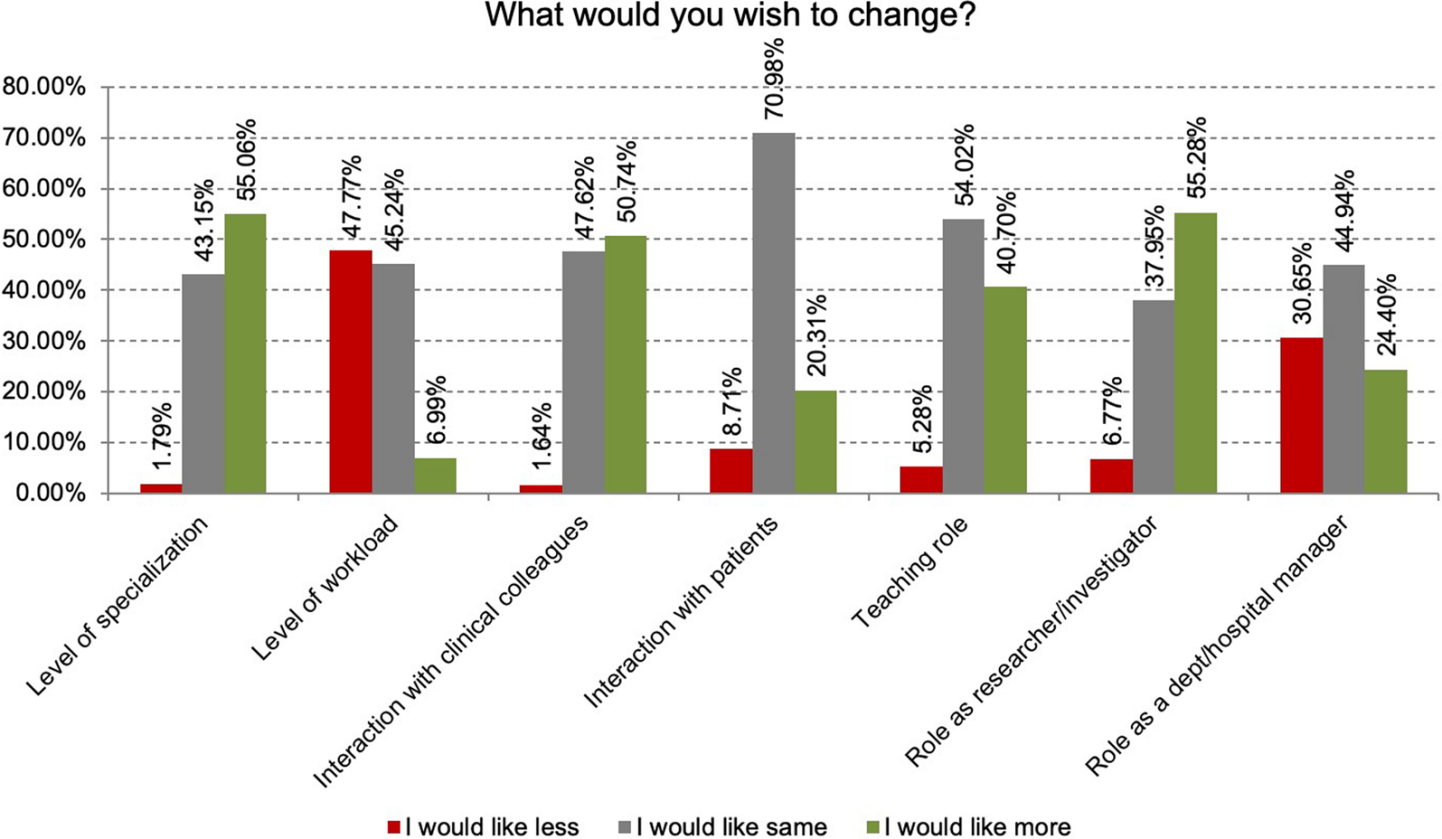
What would you wish to change? (responses *n* = 1344)

When asked what they would like to change, nearly 50% would like to reduce workload, with only 6% wanting greater workload. The level of interaction with patients, with clinical colleagues and role as a teacher was just the right amount for most, though some would prefer more (Fig. 8).

Over 50% of respondents would prefer to have a greater level of specialisation and a greater role as a researcher or investigator.

Interventional radiology was indicated as the sub-speciality area of 20% (234) of respondents (*n* = 1190).

In order to understand where interventional radiologists stand between identify their role is as a clinician versus a technician, they were asked how decisions were made concerning IR procedures: 61% stated that decisions were made jointly between the referring clinician and the IR specialist; 29% of IR specialists usually made the decision independently; whereas some 10% of respondents felt that the referring clinicians alone made the decision concerning interventional procedures. On balance, there was a greater feeling of being a clinician (weighted average of 4.01/5) versus a technician (weighted score of 2.6 of 5).

30% of IR respondents had their own outpatient clinic available; 42% would like to have one and 29% would not like an out-patient clinic. Day-case beds were available under the care of radiologists in 48%; and 30% have in-patient beds available under their care. 60% of IR respondents had IR radiology trainees working in their department.

#### Interpretation: training and practice

Radiologists see their role as highly clinical. Most radiologists consider it important to have experience in non-radiological clinical care and to have a sub-specialty expertise within radiology, despite recognising the importance of the significant general workload.

### Theme 2: About your patient and professional relationships

#### Communication with patients

76% of respondents (961/1262) regularly interact with patients face-to-face, in a variety of circumstances: 89% at the time of ultrasound, 71% while doing an interventional procedure, 52% at the time of MRI or CT examination (responses *n* = 955). Among those who do not typically communicate with patients face-to-face, 43% (129/300) would prefer having some opportunity to do so.

#### Explaining procedures to patients

Most radiologists (59%, 562/955) feel that they have sufficient time to explain procedures, though 36% feel that this is not the case.

**Fig. 9:**
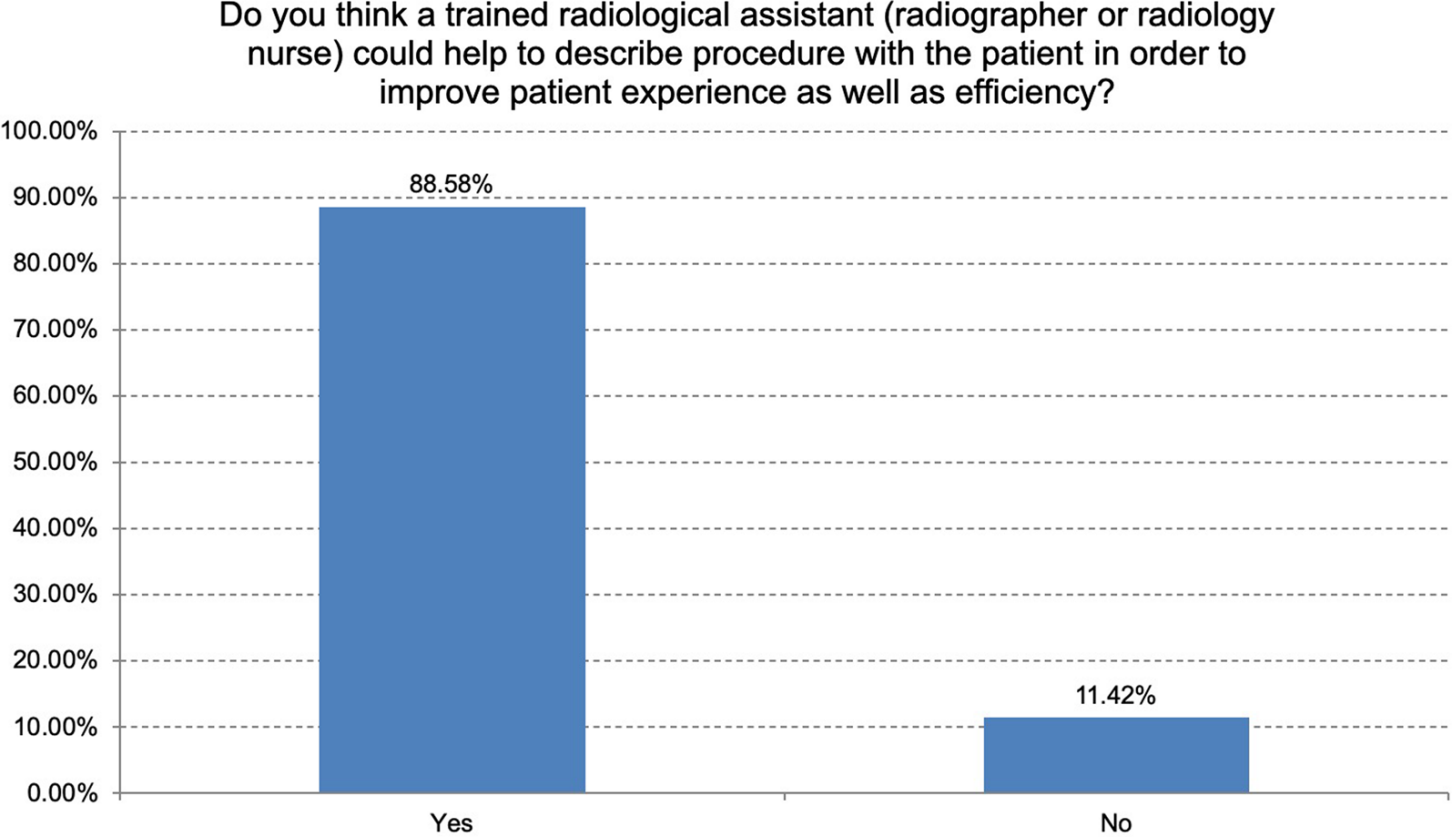
Do you think a trained radiological assistant (radiographer or radiology nurse) could help to describe procedure with the patient in order to improve patient experience as well as efficiency? (responses *n* = 946)

The ESR Patient Advisory Group proposed a question concerning whether it would be helpful to have a trained radiological assistant who could spend time with a patient in order to explain the procedure in detail and answer questions, prior to seeing the radiologist, to allow sufficient time for the patient to understand the procedure in detail, even if the radiologist has limited time. 89% of respondents felt that this might improve both the patient experience and departmental efficiency (Fig. 9). However, 11% of respondents considered this type of communication to be the domain of the medical professional only, with procedure descriptions only being appropriate if delivered by specialised or sub-specialised radiologists.

#### Providing results and breaking bad news

Although 51% have some opportunity to go through imaging results with a patient, only 25% feel that there is sufficient time to discuss radiology findings with patients, 45% sometimes have sufficient time and 24% usually do not have enough time. Over 70% do not have any specific time or reimbursement allocated in their job plans for communicating results to patients. Many respondents describe staying late or using break time/unpaid time to speak to patients when needed. Many describe this aspect of their work as important in delivering excellence of care, although others are unable to do so due to workload or due to clinicians preferring to discuss results with their patients. Only 14% of respondents have an appropriate room in their department dedicated for discussing results with patients.

Most (70%) consider that it would be useful to be able to go through images with patients in conjunction with their referring doctor. However, 30% (222 comments) feel that this would not be helpful, mainly due to a combination of logistical issues and shortage of time and staffing. There are substantial differences of opinion about who should discuss findings with the patient. Many challenges were highlighted, including difficulties arising from creating patient anxiety (particular in the case of very serious adverse findings), and inability to fully discuss treatment options (with the potential to create confusion due to different messages understood from the radiologist and the referrer). 43% feel there is potential for harm in providing findings directly to patients, though this depends on the individual patient and their outlook and wishes. Some suggest that discussion of imaging findings with the clinical team at MDT meetings is a good alternative.

Providing results to patients is usually via the referring clinician, only rarely via a secure patient portal (11%) and almost never by email. For “non-distressing” results, 61% of respondents prefer the result to be provided by the referring clinician, though this does depend on the type of investigation (e.g. US can be face-to-face); patient preference should also be considered.

Of the 57% who were happy not to discuss results with patients, 85% feel that this would be too time-consuming and 85% feel that the clinician should give the result, mainly as they can then discuss proposed treatment options. 40% feel that lack of re-imbursement is also a disincentive to committing time to direct patient interaction, with no provision in job plans for this.

**Fig. 10:**
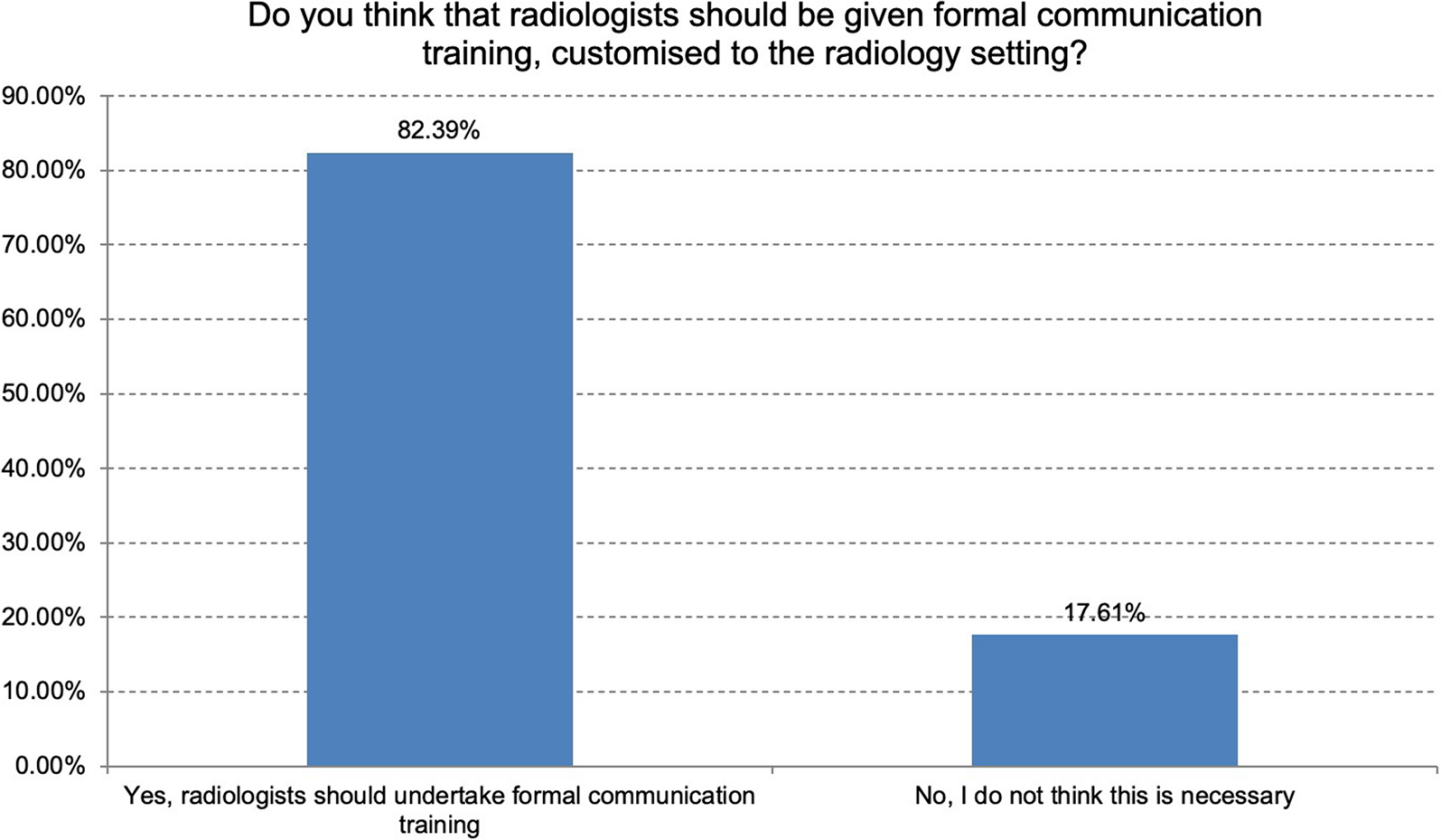
Do you think that radiologists should be given formal communication training, customised to the radiology setting? (considering that the radiologist may have limited time in the e.g. ultrasound list, they cannot go into detail with patients but perhaps should have training in “catching” the patients first shock, preparing patient for the next steps but also finding a way that makes it easier for radiologists to know how to deal with delivering bad news?) (responses *n* = 1238)

**Fig. 11:**
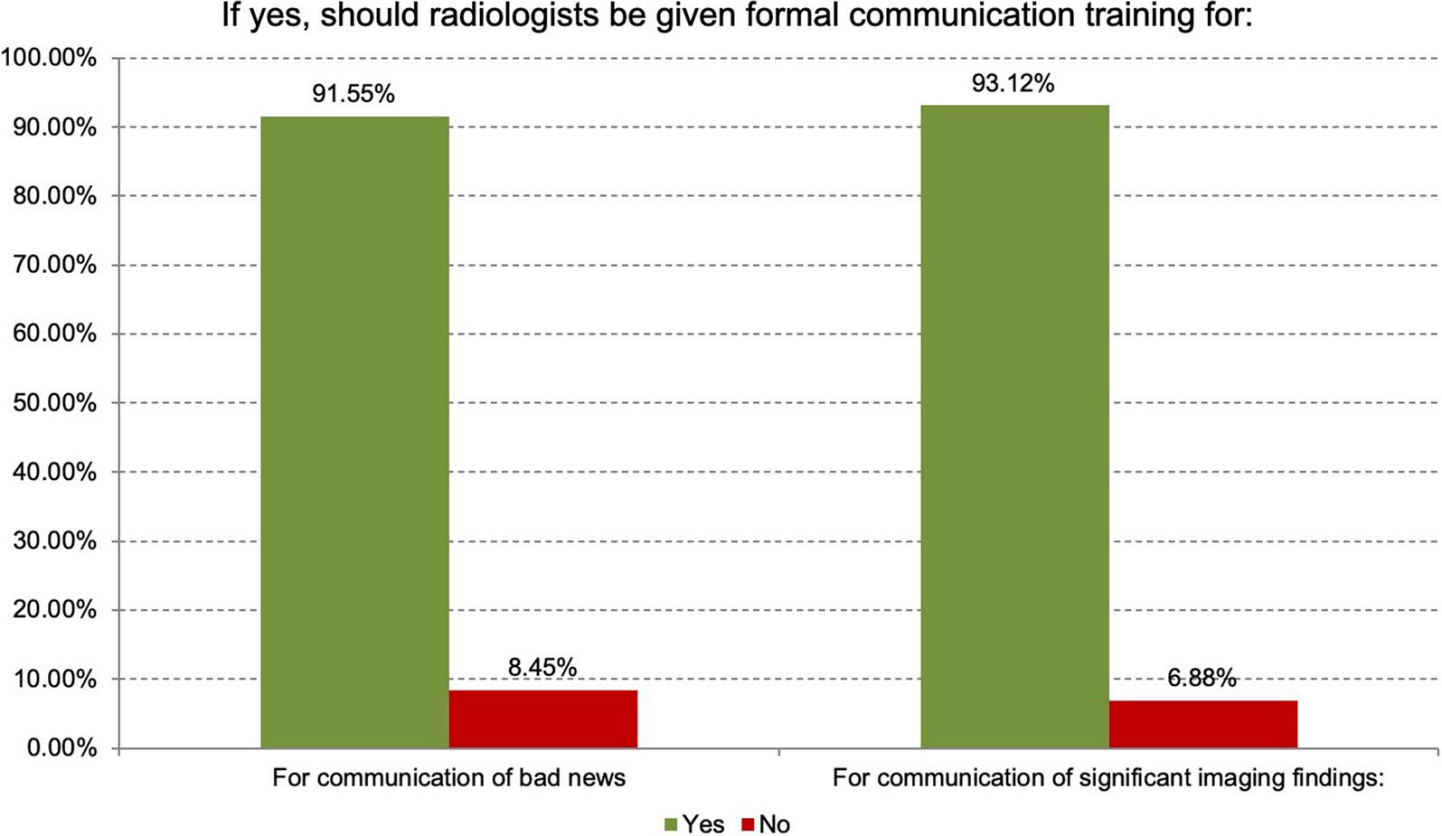
If yes, should radiologists be given formal communication training for: (responses *n* = 1018)

ESR-PAG suggested asking whether formal patient communications training had been undertaken by radiologists. Only 25% of respondents had undergone formal patient communication training, although 83% felt that radiologists should undertake such training for communication of bad news and significant imaging findings (Figs. 10, 11).

#### Patient feedback

Patient questionnaires are undertaken in 49% of departments and, in some departments, this is done regularly. Some do this in a formal way and others have a “comment book”. In some cases, patient feedback will take place in response to a particular complaint.

#### Communicating radiological errors or discrepancies

**Fig. 12:**
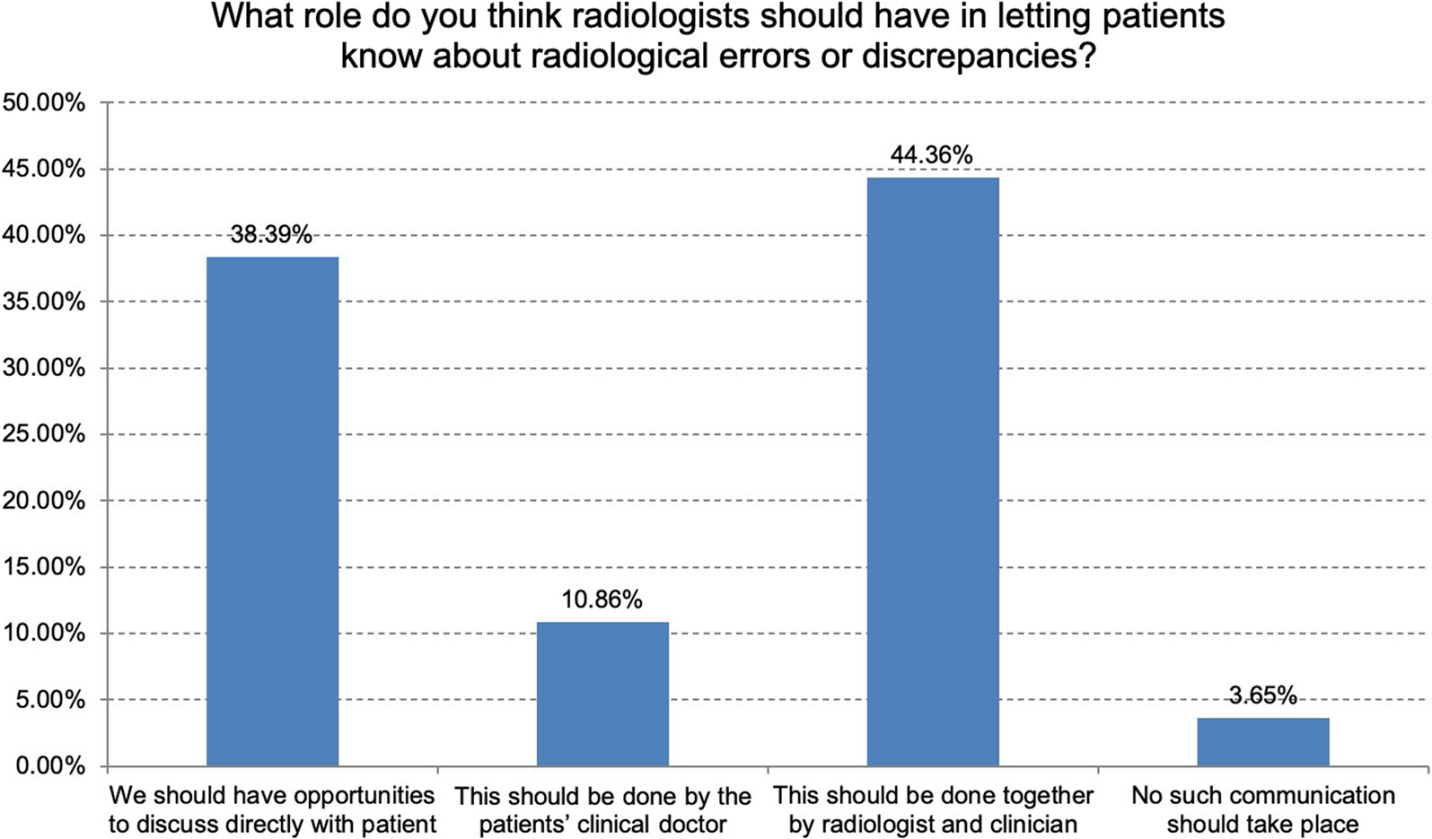
What role do you think radiologists should have in letting patients know about radiological errors or discrepancies? (responses *n* = 1206)

**Fig. 13:**
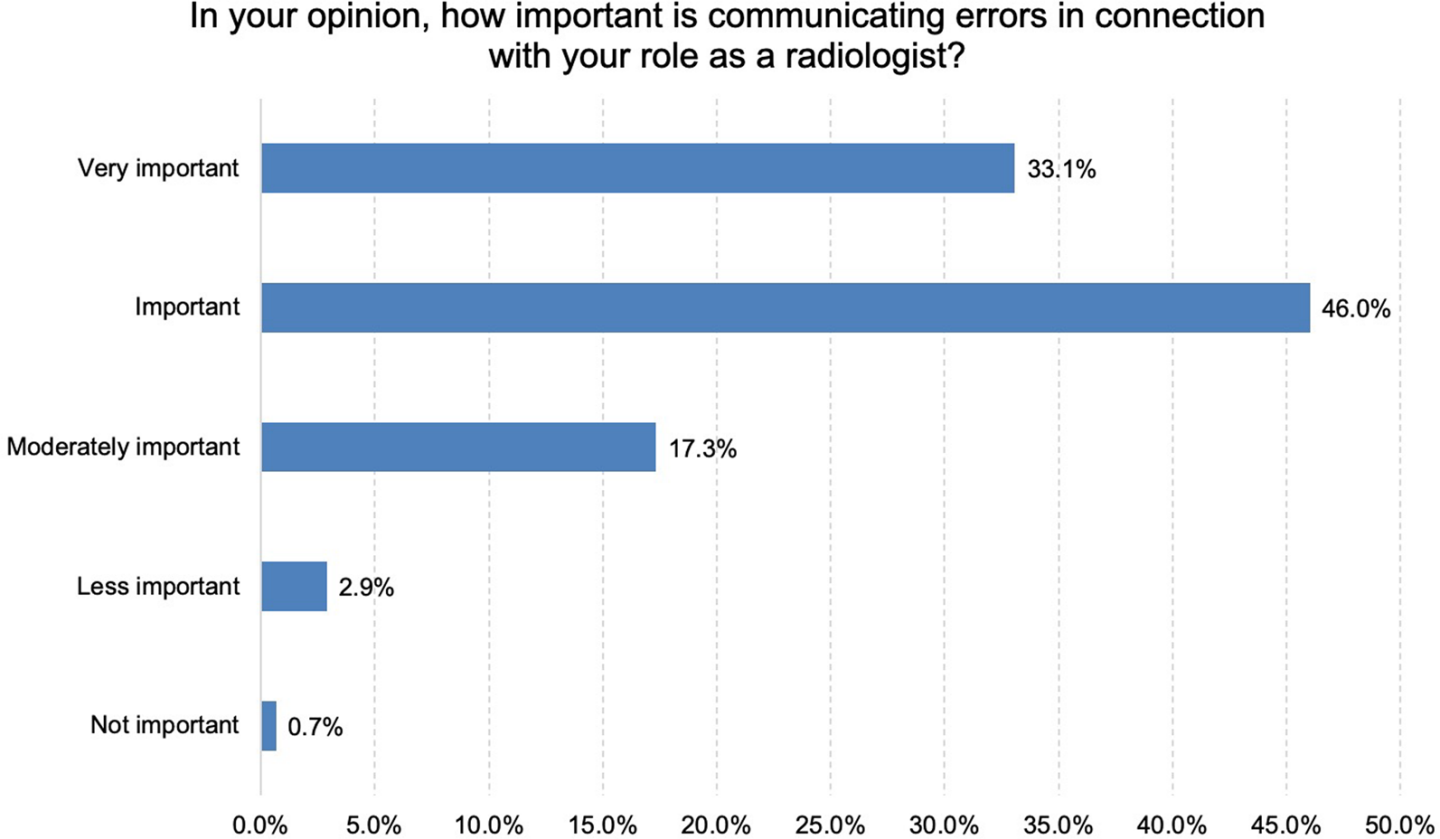
In your opinion, how important is communicating errors in connection with your role as a radiologist? (responses *n* = 1206)

Respondents were asked what role the radiologist should have in informing patients about a radiological error or discrepancy. Some commented that this depended on the type of error. The highest preference (44%) was for communication of error jointly by the radiologist and referring clinician to the patient (Figs. 12, 13). 39% felt that this should be done directly by the radiologist. 11% felt that this should be done only by the referring clinician, with the help of the radiologist if requested. Communicating errors to referring clinicians and to patients was considered important or very important (79%) and there were many comments related to this topic. This is a difficult task but essential. Review of errors was considered crucial learning and was felt to lead to becoming better specialists and humans!

#### Communications with colleagues

A majority of respondents (*n* = 1201) feel part of an organ-based clinical team (57% yes, 30% somewhat), with similar responses as to whether they feel that their clinicians see them as part of the team. 89% of respondents regularly communicate face-to-face or on the telephone with clinical colleagues and this is considered important or very important to their practice by 91%. 71% have regular MDT meetings with clinical colleagues, and for these respondents (*n* = 843), these are considered important or very important in 94%. With regards to preparing for MDTs, adequate time for preparation is available to only 55%, adequate sub-specialty staffing levels are available in 58% but re-imbursement for MDT preparation is only provided in 24% of cases.

Our role as a key player in integrating information about the patient (clinical, imaging, pathology, outcomes, follow-up) is being performed already by 56% of respondents (*n* = 1190), 38% would like the opportunity to do this more, and 6% do not feel that this is the role of the radiologist.

For the 29% in whom there were no MDT meetings (for reasons such as being in a separate location, e.g. in private practice, clinicians not having the time or MDTs not being part of standard practice), there are a variety of other practices such as informal communications when needed.

#### Visibility to patients and clinical colleagues

We are reasonably content with our visibility as radiologists to patients and within our institutions. Over 60% strongly agree that visibility is linked to being part of an organ-based speciality. There were 110 individual comments concerning our visibility to patients.

For those working in ultrasound, breast imaging and intervention, regular patient interaction allows clear visibility as a doctor involved in patient care. Some radiologists are not at all concerned about being invisible to the patients or clinicians and describe being very satisfied by providing great care to patients, even as a ‘shadow doctor’; they are not worried about how they are perceived. However, some wish to be more recognised or visible as providing an essential diagnostic service and being seen as a highly relevant part of the clinical diagnostic team, managing patient care.

Overall, 66% of respondents felt that lack of visibility to patients was a risk to radiology as a profession. We as a community may need to consider how we can improve this visibility.

#### Interpretation: patient and professional relationships

Good communication with patients is important to radiologists, though not all have this opportunity. Face-to-face communication with patients is most frequent during ultrasound and interventional procedures.

Most radiologists highly value their communications with each other and with their clinical colleagues, which is seen as a key part of the role of a radiologist. MDT meetings are identified as a conduit for good communications. However, MDT preparation and delivery is often not included in the formal job plan and making time for this role can be challenging due to limited resources.

Radiologists feel that communication of errors and learning from discrepancies and errors is a very important aspect of our professional life.

### Theme 3: Teaching, research and management

#### Teaching

**Fig. 14:**
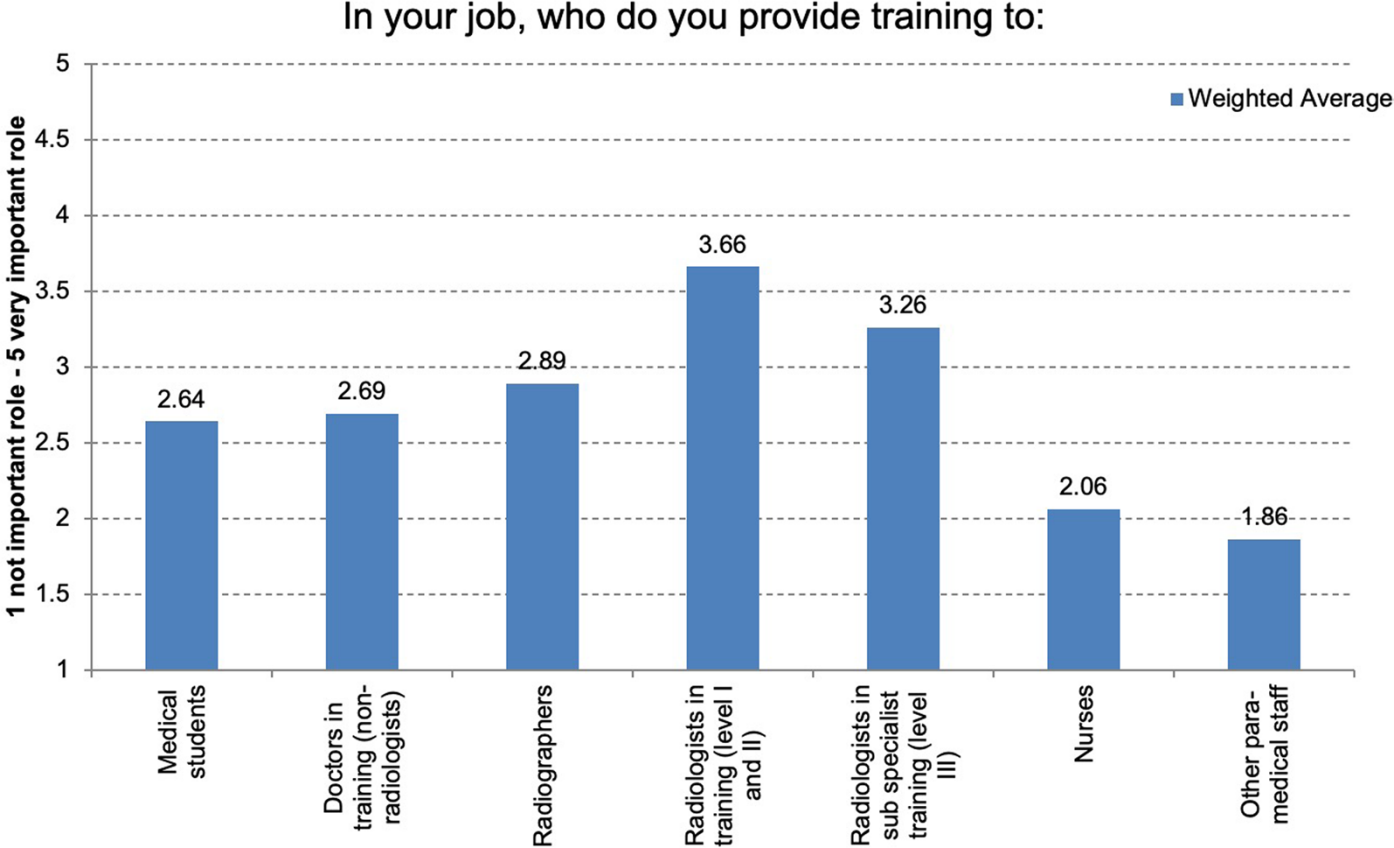
In your job, who do you provide training to: (responses *n* = 1175)

**Fig. 15:**
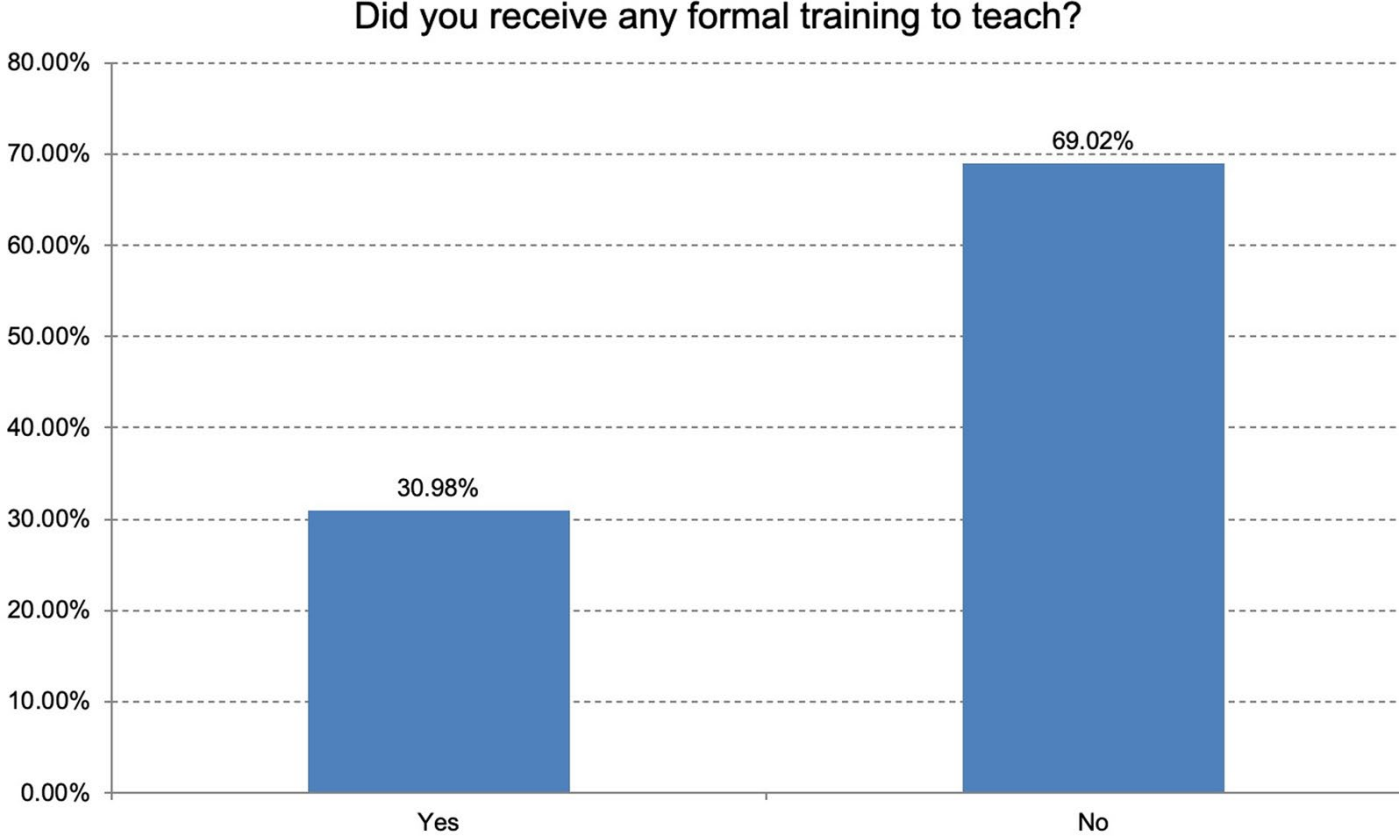
Did you receive any formal training to teach? (responses *n* = 1175)

Teaching is clearly an important role for radiologists. Training radiology residents at levels I and II is the most important provision, followed by training at level III sub-specialty radiology expertise (Figs. 14, 15). Radiologists also provide training to medical students, to junior doctors in non-radiology medical fields, and to radiographers, nurses and other para-medical staff. Most respondents (69%) have never received any formal “training to teach”, although 73% think this would be important or very important. There are a range of opinions concerning teacher training, with some responses stating that this is an innate “gift” or talent; many respondents learned “on the job” and others suggest that the quality of teaching can be improved by learning particular taught skills. Our role in teaching is considered to improve the visibility of radiologists to both the institution and to clinical colleagues (85% strongly agree or agree).

#### Research

Respondents (*n* = 1170) have a high (32%) or very high (25%) level of interest in taking part in research. However, opportunities are not always available (no opportunity in 15%, little opportunity in 39% of responses) and only 11% of respondents have many opportunities to take part in research, despite the majority working in teaching hospitals. Many (38%) would like more opportunities to contribute to data collection, and possibly to lead research. Most respondents feel that research led by radiologists is important (42%) or very important (41%) to our identity as a profession and improves our visibility to our clinical colleagues (91.5% agree or totally agree), as well as to our patients and to the media.

#### Management and service development

The head of the radiology department is always included in the hospital management board in the working environment of 36% of respondents (*n* = 1163), may be included in 32% and is not included in 22% of respondent’s departments. There are relatively limited opportunities for radiologists to influence or manage changes or developments to existing patient care pathways (10% strongly agree, 38% agree there were such opportunities), or to institutional developments (11% strongly agree, 37% agree). With regards to being involved in the development of new diagnostic or therapeutic services, radiologists felt that there were opportunities to some extent (15% agree, 50% strongly agree).

With regards to purchasing equipment in their department, 66% of radiologists had an advisory role only, with only 19% having full control over purchasing decisions, and 15% having no opportunity to provide input, with decisions being made by others.

#### Interpretation: teaching, research and management

Teaching is clearly a very important aspect of our role. Although research is an aspiration by many, the opportunities are more limited. However, radiology-led research is seen by over 90% of respondents as being important to the visibility of radiology as a profession. Radiologists are not widely included in hospital management structures or in the development of services, though opportunities do exist in some settings.

### Theme 4: Areas of delegation in your department

This optional section was completed by 70% of respondents (*n* = 809). There is a wide range of different opinions in relation to delegation of tasks to allied professionals.

However, many tasks are relatively commonly delegated to allied professionals (Table 4). The most commonly delegated procedures include checking for allergies and renal function, going through patient safety check-lists, explaining contrast injections and potential risks and checking the quality of scans prior to ending a CT or MRI examination. Peripheral venous access is delegated to an allied professional in 62% of respondent’s departments.

**Table 4:** In your department do radiographers typically: (responses *n* = 803)

	Yes		No	
Check for allergies/renal function?	88.92%	714	11.08%	89
Go through patient safety checklist for non-interventional CT and MRI?	89.04%	715	10.96%	88
Explain the contrast injection and potential risks?	80.45%	646	19.55%	157
Explain the radiation exposure and potential risk?	57.05%	457	42.95%	344
Obtain informed consent for cross-sectional imaging?	66.38%	531	33.63%	269
Perform ultrasonography?	35.21%	282	64.79%	519
Perform venous access?	62.08%	496	37.92%	303
Assess image quality autonomously before ending a CT examination?	74.81%	600	25.19%	202
Assess image quality autonomously in order to end a MR examination?	72.44%	581	27.56%	221
Assess whether or not it is justified to end/ cancel an examination?	51.19%	410	48.81%	391

**Fig. 16:**
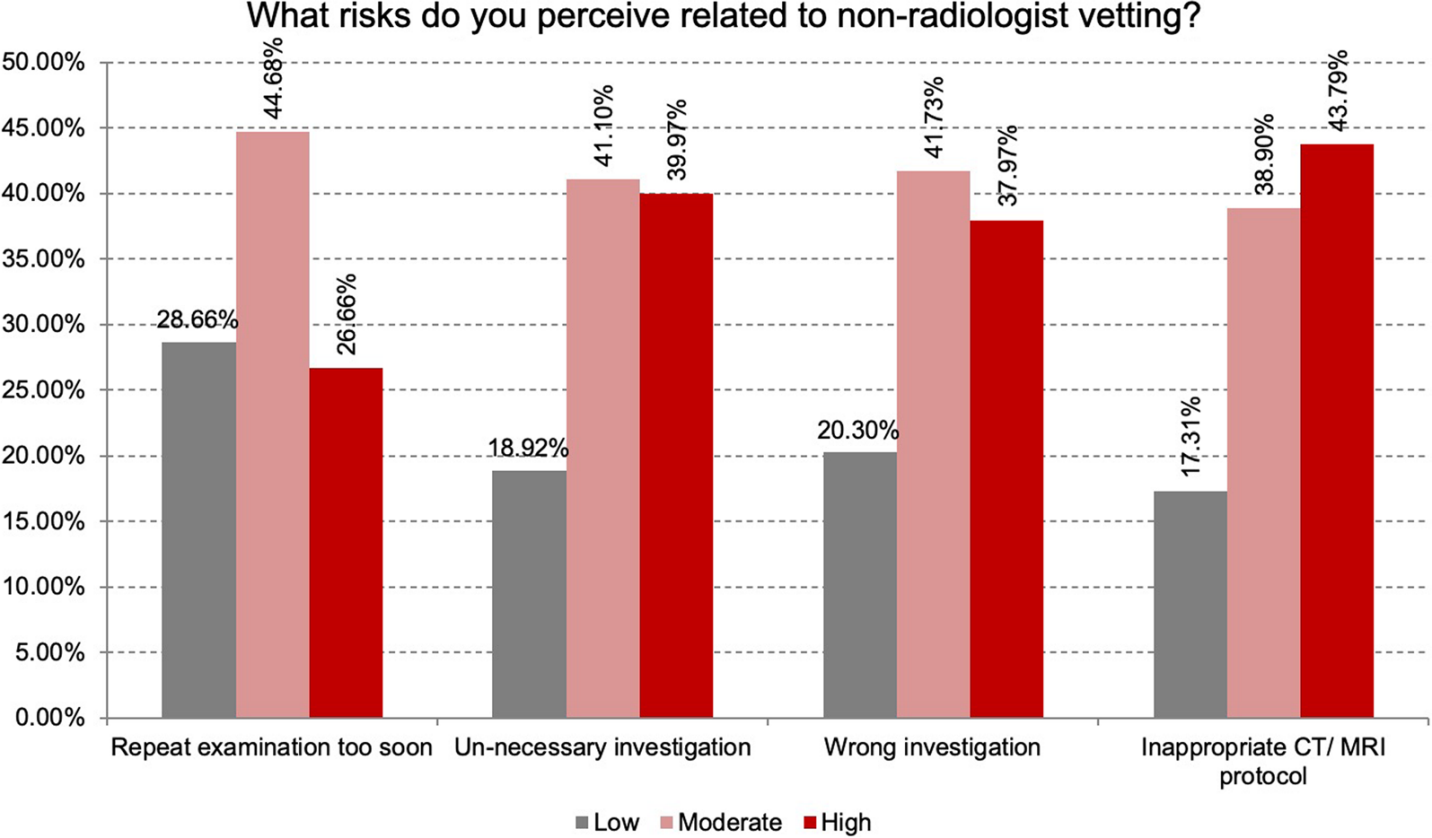
What risks do you perceive related to non-radiologist vetting? (responses *n* = 799)

With regards to checking request forms for appropriate indications (vetting), this is delegated to trained radiographers in the case of plain radiographs in 62% of responses. In the majority of cases, only the radiologist is entitled to vet indications in cross-sectional imaging, nuclear medicine or interventional radiology. In the case of vetting, 73% of respondents consider it appropriate that radiographers should have specific training for this area of delegation and there should be appropriate regulation and certification. The perceived risks of vetting by the non-radiologist are indicated in Fig. 16. The highest risk is considered to be the selection of an in-appropriate CT or MRI protocol.

## Conclusions

From the results of this survey, radiologists can understand “how we see ourselves”: our training, our practice, our communications with patients and colleagues. We can see the range of our thoughts and experiences concerning teaching, research and management and reflect on what gives us professional satisfaction, what we like about our job and what we find more difficult.

We see ourselves as highly clinical and most feel part of a dedicated clinical team. Clinical and sub-specialty training is considered very important to our role and identity, our visibility and for the future of our profession. We communicate regularly with both clinicians and patients and these communications are very important to us, in addition to giving us enjoyment in our work.

We take an active role in educating other radiologists as well as many other healthcare professionals. Many radiologists are enthusiastic to play their part in radiology research, however, opportunities are not widely available. Opportunities for management and service development are also relatively limited.

In response to the survey, what can ESR and individual radiologists do to fulfil and enhance our roles to ensure that radiology remains a desirable specialty, thereby ensuring the future success of our profession?

## Recommendations

In response to the findings of the survey, the ESR and its members can consider the following recommendations.

1. To support clinical and sub-specialty training and maintenance of competencies through its educational and accreditation activities, through Subspecialty Member Societies, European School Of Radiology (ESOR), European Diploma in Radiology (EDiR) and other ESR-endorsed subspecialty diplomas.
2. To develop patient communications strategy with recommendations for:
  enhancing patient communications training as part of ESOR/ESR coursesenhancing patient communications training as part of ESOR/ESR courses
  meeting the needs of patients with resources for patient information prior to imaging investigations and reflecting on future ways of providing results, through partnership with the ESR Patient Aadvisory Group (ESR-PAG) and National Societies Committee
  developing a framework for transparent communication and learning from errors and discrepancies.
3. To develop recommendations and guidance to support best practice in multidisciplinary team working; to enable the integration of clinical and imaging information for the benefit of patient care, whilst being mindful of pressures on limited radiology resources [1].
4. To harness the enthusiasm of radiologists by continuing to support and highlight radiology-led teaching and research opportunities through the scientific committees, meetings and publications of the ESR. These activities enhance the visibility of our speciality and are important to our identity and future of our speciality.

## Supplementary information

**Additional file 1**. Survey questions.

## Abbreviations

EDiR: European Diploma in Radiology
ESOR: European School of Radiology
ESR-PAG: ESR patient aadvisory group
MDT: Multidisciplinary team
