# Supplementary material for: The identity and role of the radiologist in 2020: a survey among ESR full radiologist members
Source: Insights Imaging. 2020 Dec 3;11:130. doi: 10.1186/s13244-020-00945-9 (PMC7711273; doi:10.1186/s13244-020-00945-9)
Supplement: Supplementary file 1 — Additional file 1. Survey questions. [file 13244_2020_945_MOESM1_ESM.pdf]

What is the identity and role of the radiologist in 2020?

Dear ESR member,

The ESR Board of Directors proposes a survey to explore the opinions of the ESR full radiologist members concerning their perceptions of the current identity and role of the radiologist.

Two basic principles can be agreed:

1. The radiologist is and will remain the responsible medical professional with regards to medical imaging diagnostics and interventional procedures performed under the auspices of radiology departments.

2. Excellence in patient care and patient safety are central to our role.

Accepting these over-arching principles, we recognise that our profession evolves as technology and healthcare evolve, giving us opportunities, as well as perhaps raising concerns in relation to our role in patient care.

Here we explore our relationship and communications with professional colleagues and patients. This may change significantly as we move into the world of instant messaging, patient portals and patient involvement.

The interaction with clinical colleagues may be challenged in relation to turf battles and whether we see ourselves (and are seen) as technicians or as clinicians. Imaging and imaging-guided procedures are integral to medical education, research and healthcare management. What is our role in these important areas to ensure the development of high-quality services and care?

Delegation of some aspects of work to allied healthcare professionals has become part of teamwork and radiology is not immune. What are radiologists' opinions concerning skill mix in radiology?

This survey attempts to explore radiologists' opinions in relation to their identity with four main sections:

1. **About you, your clinical role and your training.**
2. **About your role in patient and professional relationships.**
3. **About your role in education, research and management.**
4. **About delegation: what happens in your department and what is your opinion?**

This is an in-depth survey and as such we understand it is somewhat longer, but seeing the importance of this topic, we kindly ask you to please take the time and provide us with your valuable input. The survey should take approximately 25-30 minutes and its results will be discussed during the ESR National Societies Committee meeting which will be held at the ECR 2020.

In order to express our appreciation for your support, we will offer 10 free registrations to ECR 2020 in

a prize draw.

The winners will be selected randomly among those who completed the survey (at least mandatory sections).

Many thanks for your help!

Kind regards,
Andrea Rockall
Chairperson of the ESR National Societies Committee

What is the identity and role of the radiologist in 2020?

About you, your clinical role and your training

1. If you would like to participate in the prize draw (free ECR 2020 registration), please let us know your email address.

* 2. In which country do you practice?

* 3. How many years have you worked as an accredited radiologist (after completion of specialist training)?

- 0-5
- 6-10
- 11-15
- 16-20
- >20

* 4. What is your age?

- 25-34 years
- 35-44 years
- 45-54 years
- 55-64 years
- Age 65 or older

5. What is your gender?

- Male
- Female
- Prefer not to say

* 6. How would you best describe your primary practice setting?

	Academic teaching hospital	Non-university or district general hospital with teaching	District general hospital (i.e. smaller/community hospital) without teaching	Private practice	Retired
Primary practice setting					

* 7. How would you best describe your secondary practice setting?

	Not applicable	Academic teaching hospital	Non-university or district general hospital with teaching	District general hospital without teaching	Private practice	Retired
Additional practice setting						

* 8. Does your practice setting offer:

(flexible working: could include early/late starts/ rotating days working)
(part-time: less than standard departmental full-time contract)

	Yes	No	Don't know
Flexible working for men			
Flexible working for women			
Part-time working for men			
Part-time working for women			

* 9. How would you best describe the imaging modalities of your own personal practice?

	None	Low	Moderate	High	Very High
Plain film	◯	◯	◯	◯	◯
Mammography	◯	◯	◯	◯	◯
Ultrasound	◯	◯	◯	◯	◯
Radiographic screening	◯	◯	◯	◯	◯
CT	◯	◯	◯	◯	◯
MRI	◯	◯	◯	◯	◯
Nuclear medicine	◯	◯	◯	◯	◯
Interventional procedures (general)	◯	◯	◯	◯	◯
Interventional procedures (sub-specialist)	◯	◯	◯	◯	◯

* 10. How would you best describe your own "organ-based or clinical" areas of practice?

	None	Low	Moderate	High	Very High
General	◯	◯	◯	◯	◯
Neuro	◯	◯	◯	◯	◯
Head and Neck	◯	◯	◯	◯	◯
Pulmonary	◯	◯	◯	◯	◯
Cardiac	◯	◯	◯	◯	◯
Breast	◯	◯	◯	◯	◯
Gastrointestinal	◯	◯	◯	◯	◯
Genitourinary	◯	◯	◯	◯	◯
Musculoskeletal	◯	◯	◯	◯	◯
Paediatric Imaging	◯	◯	◯	◯	◯
Oncology imaging	◯	◯	◯	◯	◯
Vascular intervention	◯	◯	◯	◯	◯
Non-vascular intervention	◯	◯	◯	◯	◯

* 11. How do you rate your enjoyment of different aspects of your job?

	Strongly disagree	Disagree	Neutral	Agree	Strongly agree	Not applicable
Being a general radiologist						
Being a specialized radiologist						
Interaction with colleagues in the radiology department						
Interaction with clinical colleagues						
Involvement in multidisciplinary meetings/tumour board						
Interaction with patients						
Teaching						
Service evaluation and audit						
Performing research						
Involvement with hospital management						

* 12. What would you wish to change?

	I would like less	I would like same	I would like more
Level of specialization			
Level of workload			
Interaction with clinical colleagues			
Interaction with patients			
Teaching role			
Role as researcher/investigator			
Role as a dept/hospital manager			

* 13. What is the level of “visibility” of radiologists in your institution? *(Visibility could include for example the recognition of our role in patient management, hospital management or to patient groups)*

- High
- Medium
- Low

* 14. Are you happy with the level of visibility of radiologists in your institution?

	Yes	Medium	No
With patients	◯	◯	◯
With clinicians	◯	◯	◯
With institution	◯	◯	◯

* 15. In your opinion, is visibility important for our identity in the clinical service e.g. is it important for radiologists to be a visible member of organ-based clinical service?

Strongly disagree	Disagree	Neutral	Agree	Strongly agree
★	★	★	★	★

* 16. In your institution:

	Yes	No	Not Applicable	I don't know
Radiologists' names and/or expertise are listed on the hospital website	◯	◯	◯	◯
Radiologists are listed as part of the specialist clinical care teams on website or letterhead	◯	◯	◯	◯

What is the identity and role of the radiologist in 2020?

About your training

* 17. Did you undertake a period of clinical training?

◯ Yes

◯ No

What is the identity and role of the radiologist in 2020?

* 18. At what stage of your training was this done?

☐ As undergraduate (medical student)

☐ As clinical resident

☐ As radiology resident

* 19. For how long altogether?

- 3 months
- 6 months
- 9 months
- 12 months
- 12-24 months
- > 24 months

What is the identity and role of the radiologist in 2020?

* 20. Do you think radiologists should have a mandatory period of non-radiology clinical experience, either before or during radiology training?

- Yes
- No

What is the identity and role of the radiologist in 2020?

* 21. If yes, for how long?

- 3 to 6 months
- 9 to 12 months
- 12 to 24 months
- > 24 months

What is the identity and role of the radiologist in 2020?

* 22. Did you undertake a period of clinical or experimental research?

- Yes (formal, e.g. Master's degree or PhD)
- Yes (informal)
- No

What is the identity and role of the radiologist in 2020?

* 23. If yes, for how long?

- 3 to 6 months
- 9 to 12 months
- 12 to 24 months
- > 24 months

* 24. Was this a formal requirement of your training programme?

- Yes
- No

What is the identity and role of the radiologist in 2020?

* 25. Did you undertake a period of subspecialist training/clinical fellowship?

- Yes
- No

What is the identity and role of the radiologist in 2020?

* 26. If yes, for how long?

- 3 to 6 months
- 9 to 12 months
- 12 to 24 months
- > 24 months

* 27. Did you undertake the period of subspecialist training/clinical fellowship:

- In your own training department
- In a different department

What is the identity and role of the radiologist in 2020?

* 28. In your opinion, do you think it is important for radiologists to have an area of subspecialisation?

- ◯ No, general work is most in demand
- ◯ Yes

Comment

* 29. Do you think that having a sub-speciality accreditation improves your identity as a radiologist?

	Strongly disagree	Disagree	Neutral	Agree	Strongly agree
To your own perception	◯	◯	◯	◯	◯
To your clinical colleagues	◯	◯	◯	◯	◯
To patients	◯	◯	◯	◯	◯

* 30. Do you have informatics training?

- ◯ None
- ◯ Basic understanding
- ◯ Basic skills and training
- ◯ Advanced skills and training, including coding/AI developments

What is the identity and role of the radiologist in 2020?

About your role in patient and professional relationships

* 31. How important do you think the role of the radiologist is in ensuring that patient experience is satisfactory in your department?

Not important	Less important	Moderately important	Important	Very important
☆	☆	☆	☆	☆

* 32. Do you think that increasing visibility with patients would change your identity as a radiologist?

	Strongly disagree	Disagree	Neutral	Agree	Strongly agree
I would feel more like a clinician	○	○	○	○	○
The patient would see me as a clinical doctor	○	○	○	○	○
I would feel more valued as part of the patient care delivery	○	○	○	○	○

* 33. In your opinion, how important to your job satisfaction is it for you to be seen by patients as part of the clinical care team?

Not important	Less important	Moderately important	Important	Very important
☆	☆	☆	☆	☆

Comment

* 34. Do you feel that lack of visibility to patients is a risk to radiology as a profession?

- ○ Yes
- ○ No

Comment

* 35. Do you regularly communicate with patients clinically (face to face) in your practice?

- ○ Yes
- ○ No

What is the identity and role of the radiologist in 2020?

IF YES (I do regularly communicate with patients face to face)

* 36. Do you see patients face to face?

	Yes	No
In ultrasound?	◯	◯
For interventional procedures?	◯	◯
In MR or CT unit?	◯	◯
To go through imaging results?	◯	◯

Other (please comment)

* 37. Do you typically have sufficient time to discuss the procedure with the patient?

- ◯ Yes
- ◯ No
- ◯ Not applicable

* 38. Do you typically have sufficient time to discuss the radiology findings with the patient?

- ◯ Yes
- ◯ Sometimes
- ◯ Usually not
- ◯ Not applicable

* 39. Do you have specific time for this in your job plan or is it not reimbursed (or provided for) in your system?

- ◯ Yes
- ◯ No
- ◯ Not applicable

Other (please comment)

* 40. Is there a specific/dedicated clinic room available in your department to discuss radiology findings?

- Yes - highly adequate
- Yes - not really adequate
- No
- Not applicable

* 41. Do you think it would be useful to be in the outpatient clinic or clinical department together with the clinicians to go through images with the patient?

- Yes
- No

What is the identity and role of the radiologist in 2020?

42. If no, why not? Please indicate briefly in comment box.

What is the identity and role of the radiologist in 2020?

* 43. Do you think a trained radiological assistant (radiographer or radiology nurse) could help to describe procedure with the patient in order to improve patient experience as well as efficiency?

- Yes
- No

Comment

What is the identity and role of the radiologist in 2020?

IF NO (I do not regularly communicate with patients face to face

* 44. If you do not typically see/communicate with patients face to face (e.g. mainly reporting on PACS), would you prefer seeing the patients more and discussing their images/findings with them?

- ◯ Yes
- ◯ No

What is the identity and role of the radiologist in 2020?

* 45. If not, would you prefer not to see patients because:

	Yes	No
Too time consuming	◯	◯
This would not be reimbursed	◯	◯
I think the clinician should give the result	◯	◯

Comment

What is the identity and role of the radiologist in 2020?

* 46. Do you think providing the findings directly to the patient would be beneficial?

- ◯ Yes
- ◯ No

Comment

* 47. Do you think providing the findings directly to the patient would be harmful?

- ◯ Yes
- ◯ No

Comment

What is the identity and role of the radiologist in 2020?

* 48. If yes, why?

- [ ] Could be distressing
- [ ] No relationship with patient
- [ ] Not able to discuss onward treatment therefore patient is left wondering what will happen
- [ ] Other (please comment)

What is the identity and role of the radiologist in 2020?

* 49. Have you had any formal patient communication training?

- ○ Yes
- ○ No

* 50. Do you think that radiologists should be given formal communication training, customised to the radiology setting? *(considering that the radiologist may have limited time in the e.g. ultrasound list, they cannot go into detail with patients but perhaps should have training in "catching" the patients first shock, preparing patient for the next steps but also finding a way that makes it easier for radiologists to know how to deal with delivering bad news?)*

- ○ Yes, radiologists should undertake formal communication training
- ○ No, I do not think this is necessary

What is the identity and role of the radiologist in 2020?

* 51. If yes, should radiologists be given formal communication training for:

	Yes	No
For communication of bad news	○	○
For communication of significant imaging findings:	○	○

What is the identity and role of the radiologist in 2020?

* 52. How are diagnostic findings (“non-distressing” for example non-life limiting conditions such as gallstones or degenerative joint) provided to the patient in your institution?

	Never	Sometimes	Often	Usually
To patient in the department face to face	○	○	○	○
Via email to the patient	○	○	○	○
Via secure patient portal	○	○	○	○
To referring clinican	○	○	○	○

Comment

* 53. In your opinion, how would you prefer to provide “non-distressing” patient findings?

	Never	Sometimes	Often	Usually
To patient in the department face to face	○	○	○	○
Via email to the patient	○	○	○	○
Via secure patient portal	○	○	○	○
To referring clinician	○	○	○	○

Comment

* 54. Does your department undertake patient feedback questionnaires?

- ○ Yes
- ○ No
- ○ I don't know

Comment

What is the identity and role of the radiologist in 2020?

* 55. What role do you think radiologists should have in letting patients know about radiological errors or discrepancies?

- ◯ We should have opportunities to discuss directly with patient
- ◯ This should be done by the patients’ clinical doctor
- ◯ This should be done together by radiologist and clinician
- ◯ No such communication should take place
- ◯ Comment

* 56. In your opinion, how important is communicating errors in connection with your role as a radiologist?

Not important	Less important	Moderately important	Important	Very important
★	★	★	★	★

Comment

What is the identity and role of the radiologist in 2020?

Interaction with clinicians

* 57. Do you feel part of one or more particular ‘clinical team’ such as a specialist service (e.g. Gastrointestinal cancer, neuro, musculoskeletal etc).

- ◯ Yes
- ◯ Somewhat
- ◯ No

* 58. In your opinion, do your clinical colleagues think of you as being part of their clinical team?

- ◯ Yes
- ◯ Somewhat
- ◯ No

* 59. Do you regularly provide face to face or telephone opinions to your clinical colleagues?

- Yes
- No

What is the identity and role of the radiologist in 2020?

* 60. If yes, how important is this to your practice?

Not important	Less important	Moderately important	Important	Very important
★	★	★	★	★

What is the identity and role of the radiologist in 2020?

* 61. If not, how important do you think this could be to your practice?

Not important	Less important	Moderately important	Important	Very important
★	★	★	★	★

What is the identity and role of the radiologist in 2020?

* 62. Do you have regular formally scheduled weekly meetings (eg, multidisciplinary case reviews, tumour boards) with the clinical colleagues?

- Yes
- No

What is the identity and role of the radiologist in 2020?

63. If you do not have regular weekly meetings with the clinical colleagues, what is the reason?

- ◯ No time to attend due to heavy workload
- ◯ Not reimbursed
- ◯ Other (please comment)

What is the identity and role of the radiologist in 2020?

* 64. If yes, in which form?

- ◯ In the form of tumour board/ multi-disciplinary team meetings
- ◯ Informal
- ◯ Both (tumour boards/ multi-disciplinary team meetings and informal)

* 65. How important are these to your practice?

Not important	Less important	Moderately important	Important	Very important
☆	☆	☆	☆	☆

* 66. In which form are multidisciplinary meetings recognised by your hospital/ department management as an essential part of the radiologists' work?

	Yes	No
Adequate time provided for preparation and review of external images?	◯	◯
Adequate staffing and subspecialist competence (level III training) provided?	◯	◯
Regularly reimbursed service in the budget?	◯	◯

What is the identity and role of the radiologist in 2020?

* 67. Do you see your role as important in integrating information about the patient (clinical, imaging, pathology, outcome, follow-up)?

- Yes: I do this already
- Yes: I would like to do this more
- No: I do not think this is the role of the radiologist

* 68. Are you a sub-specialist interventional radiologist?

- Yes
- No

What is the identity and role of the radiologist in 2020?

In relation to interventional procedures

69. Who typically decides on the procedure in your department?

- Radiologist
- Referring clinician
- Together

70. In your department, do you feel you work more as a

	Strongly disagree	Disagree	Neutral	Agree	Strongly agree
Clinician					
Technician					

71. Do you feel that less experienced Interventional Radiology (IR) practitioners may at times feel under pressure to perform a procedure if the referring clinician wants it?

Strongly disagree	Disagree	Neutral	Agree	Strongly agree
★	★	★	★	★

72. Would you like to have your own outpatient clinic?

- I have one
- Yes, I would like to have one
- No

73. Do you have radiology day case or in-patient beds available for your use?

	Not available but *would not like*	Not available but *would like to have*	Already available
Day case beds	◯	◯	◯
In patient beds	◯	◯	◯

74. In your department are there junior Interventional Radiology (IR) trainees who are training to become sub-specialty IR radiologists?

◯ Yes

◯ No

What is the identity and role of the radiologist in 2020?

About your role in education, research, management and service development

* 75. In your job, who do you provide training to:

	Not important role	Less important role	Moderately important	Important role	Very important role
Medical students	◯	◯	◯	◯	◯
Doctors in training (non-radiologists)	◯	◯	◯	◯	◯
Radiographers	◯	◯	◯	◯	◯
Radiologists in training (level I and II)	◯	◯	◯	◯	◯
Radiologists in sub specialist training (level III)	◯	◯	◯	◯	◯
Nurses	◯	◯	◯	◯	◯
Other para-medical staff	◯	◯	◯	◯	◯

* 76. Did you receive any formal training to teach?

◯ Yes

◯ No

* 77. How important do you think formal teacher training is?

Not important	Less important	Moderately important	Important	Very important
☆	☆	☆	☆	☆

Comment

* 78. Do you think that a teaching role improves the visibility and identity of radiologists?

	Strongly disagree	Disagree	Neutral	Agree	Strongly agree
To the institution	○	○	○	○	○
To clinical colleagues	○	○	○	○	○
To patients	○	○	○	○	○
To public	○	○	○	○	○

* 79. Do you think radiological training should include a brief training on Artificial Intelligence (AI) computing technology and/or software engineering skills?

- ○ Yes - Basic training (understanding concepts) should be included in core curriculum.
- ○ Yes - Advanced training (ability to undertake) focussed on radiologists should be available
- ○ No

What is the identity and role of the radiologist in 2020?

With regards to research

* 80. What is your level of interest in taking part in radiological research?

No interest	Low interest	Medium	High interest	Very high interest
☆	☆	☆	☆	☆

* 81. Are there opportunities to take part in research in your dept.?

No opportunities	Little	Enough	Several	Many opportunities
☆	☆	☆	☆	☆

* 82. Would you like more opportunities to contribute to data collection for research but not lead research?

- [ ] No
- [ ] Perhaps
- [ ] Yes

* 83. In your opinion, is research led by radiologists important to our identity as a profession?

Not important	Less important	Moderately important	Important	Yery important
★	★	★	★	★

* 84. Research led by radiologists improves the visibility and identity of radiologists

	Totally disagree	Disgaree	Neutral	Agree	Totally agree
To clinical colleagues	○	○	○	○	○
To patients	○	○	○	○	○
To media	○	○	○	○	○

* 85. In your opinion, do you think research should be part of radiology training?

	Strongly disagree	Disagree	Neutral	Agree	Strongly agree
Basic understanding (methods, statistics and ethics)	○	○	○	○	○
Direct involvement in a research project	○	○	○	○	○

What is the identity and role of the radiologist in 2020?

Management-related questions with regards to <u>your</u> institution

* 86. Is the head of radiology departments included in the Hospital Management / Medical Board?

- ○ Yes
- ○ No
- ○ Not automatically, but in some instances
- ○ I don't know

* 87. Do radiologists have opportunities to develop senior management roles to influence patient treatment pathway?

Strongly disagree	Disagree	Neutral	Agree	Strongly agree
★	★	★	★	★

* 88. Do radiologists have opportunities to develop senior management roles to influence institutional development?

Strongly disagree	Disagree	Neutral	Agree	Strongly agree
★	★	★	★	★

* 89. Do radiologists have opportunities to develop new diagnostic services?

Strongly disagree	Disagree	Neutral	Agree	Strongly agree
★	★	★	★	★

* 90. Do radiologists have opportunities, with clinical colleagues, to develop new therapeutic services?

Strongly disagree	Disagree	Neutral	Agree	Strongly agree
★	★	★	★	★

* 91. How much discretion do radiologists have in your department to purchase equipment?

- ○ Full control
- ○ Advisory role only
- ○ No input (decisions made by others)

What is the identity and role of the radiologist in 2020?

92. Would you like to answer the optional section "Areas of delegation: What happens in your department and what is your opinion" (13 more questions) or proceed to the end to submit your answers?

- ○ Proceed to the optional section "Areas of delegation"
- ○ Proceed to the end to submit answers

What is the identity and role of the radiologist in 2020?

Areas of delegation: What happens in your department and what is your opinion? (Optional section)

In this section, we would like to know your opinion on the delegation of some roles traditionally reserved for radiologists to other allied professionals.

93. In your department do radiographers typically:

	Yes	No
Check for allergies/renal function?	○	○
Go through patient safety checklist for non-interventional CT and MRI?	○	○
Explain the contrast injection and potential risks?	○	○
Explain the radiation exposure and potential risk?	○	○
Obtain informed consent for cross-sectional imaging?	○	○
Perform ultrasonography?	○	○
Perform venous access?	○	○
Assess image quality autonomously before ending a CT examination?	○	○
Assess image quality autonomously in order to end a MR examination?	○	○
Assess whether or not it is justified to end/ cancel an examination?	○	○

94. In your department currently, is **vetting** (checking if the indication is appropriate) of radiology requests always done by an accredited radiologist or is there some delegation to trained radiographic staff?

	Only radiologist	May be delegated to trained radiographer
Plain film	○	○
Ultrasound (US)	○	○
CT	○	○
MRI	○	○
Nuclear Medicine	○	○
Interventional Radiology (IR)	○	○

95. If radiographers undertake some vetting in your department, do you think the training for this should be formally regulated/certified?

- Yes
- No
- Not applicable

96. What risks do you perceive related to non-radiologist vetting?

	Low	Moderate	High
Repeat examination too soon			
Un-necessary investigation			
Wrong investigation			
Inappropriate CT/ MRI protocol			

97. In your department currently, is **selection of imaging protocol** of radiology requests always done by an accredited radiologist or is there some delegation to trained radiographic staff?

	Only radiologist	May be delegated to trained radiographer	Not done
Plain film			
Ultrasound (US)			
CT			
MRI			
Nuclear Medicine			
Interventional Radiology (IR)			

98. Are radiographers in your department trained in terminology related to anatomy and pathology?

- Yes
- No

99. Do allied professionals (radiographers) issue imaging reports in your department?

- Yes
- Limited reporting
- Never

100. Do you agree with this in principle?

- ◯ Yes
- ◯ No

101. Who should have final decision of whether reporting radiographer is acceptable in department?

- ◯ Head of department(radiologist or other medically trained head of radiology)?
- ◯ Hospital management team?

102. What level of performance metric do you think would be acceptable for radiographic reporting?

- ◯ Audited findings within the same range as between two expert radiologists
- ◯ Audited findings within 10% of expert radiologist
- ◯ Audited findings within the same range as training radiologist
- ◯ Not applicable

103. Who should take responsibility for significant errors in radiographic reports?

- ◯ Radiographer
- ◯ Hospital management
- ◯ Radiologists

104. What do you perceive are the drivers for radiographer skill-mix/delegation?

	Strongly disagree	Disagree	Neutral	Agree	Strongly agree	Not applicable / I don't know
Retention of excellent staff by increasing opportunities for professional development	◯	◯	◯	◯	◯	◯
Retention of staff by increasing responsibility and therefore pay enhancement/ improved professional status	◯	◯	◯	◯	◯	◯
Work-load pressure on radiologists	◯	◯	◯	◯	◯	◯
Insufficient provision of radiologist numbers	◯	◯	◯	◯	◯	◯
Backlog reporting	◯	◯	◯	◯	◯	◯

Others (please comment)

105. What do you see as the main disadvantages of reporting by radiographers?

- ☐ De-skilling of junior radiologists/leading to de-skilling in the longer term - e.g. in plain film reporting
- ☐ The need to train radiographers and ensure quality metrics are met/audit
- ☐ Reports are not actionable - may end up being re-scanned or re-reported by radiologist
- ☐ May create more work - e.g. gynae ultrasound scanning in general practitioner's (GP) clinics led to huge increase in MRI of simple cysts/non worrisome cysts - as the sonographer was not confident to call benign (unintended consequences)
- ☐ Less radiologists trained
- ☐ The fact that radiographer reports are not provided by someone with full medical training
- ☐ Other (please comment)

What is the identity and role of the radiologist in 2020?

Thank you very much for your time!

Please select Previous to go back to the survey or Done to submit your replies.
